# Development and internal validation of a radiomics-clinical combined model for predicting axillary pathological complete response in clinically node-positive breast cancer patients after neoadjuvant chemotherapy

**DOI:** 10.3389/fonc.2026.1876446

**Published:** 2026-07-10

**Authors:** Weitao Yan, Wenxuan Lu, Ying Dai, Xiangchao Meng, Kai Feng

**Affiliations:** 1 Breast Disease Diagnosis and Treatment Center, The First Hospital of Qinhuangdao, Qinhuangdao, Hebei, China; 2 Department 2 of Respiratory and Critical Care Medicine, The First Hospital of Qinhuangdao, Qinhuangdao, Hebei, China

**Keywords:** axillary pathological complete response, breast cancer, neoadjuvant chemotherapy, nomogram, prediction model, radiomics

## Abstract

**Background:**

Accurate prediction of axillary pathological complete response (apCR) after neoadjuvant chemotherapy (NAC) in clinically node-positive (cN+) breast cancer patients may guide surgical de-escalation from axillary lymph node dissection to sentinel lymph node biopsy. This study aimed to develop and internally validate a combined radiomics-clinical prediction model for apCR.

**Methods:**

This single-center retrospective study enrolled 386 cN+ breast cancer patients (training, n = 270; validation, n = 116). Pre-NAC DCE-MRI radiomic features were extracted from primary tumors. LASSO regression selected eight features for Rad-score construction. Clinical predictors were identified via logistic regression. Model performance was evaluated using AUC, calibration metrics, and decision curve analysis.

**Results:**

The overall apCR rate was 43.5% (168/386). The combined model (tumor size, HER2 status, Ki-67, breast clinical complete response, and Rad-score) achieved a validation AUC of 0.703 (95% CI, 0.610–0.792). It significantly outperformed the radiomics-only model (ΔAUC = 0.094, P = 0.004) but not the clinical-only model (ΔAUC = 0.020, P = 0.713). The combined model showed a calibration slope of 0.811 and an intercept of 0.018, indicating moderate overfitting. Risk stratification showed monotonic gradients across tertiles (low 18.8%, intermediate 48.0%, high 58.8%). After bootstrap bias correction, the optimism-corrected training AUC of the combined model was 0.742.

**Conclusions:**

The combined model demonstrated moderate discriminatory ability but did not add significant value over clinical predictors alone. The current misclassification rate precludes direct clinical application for surgical de-escalation. External multicenter validation is warranted.

## Introduction

Neoadjuvant chemotherapy (NAC) has become the standard treatment for locally advanced and clinically node-positive breast cancer, achieving pathological complete response in a substantial proportion of patients (1, 2). Among patients with clinically node-positive (cN+) disease, axillary pathological complete response (apCR, defined as ypN0) occurs in approximately 35–55% of cases depending on molecular subtype, with higher rates observed in human epidermal growth factor receptor 2 (HER2)-positive and triple-negative breast cancer (TNBC) subtypes (3, 4). The current standard surgical management following NAC includes axillary lymph node dissection (ALND), which carries significant morbidity including lymphedema, shoulder dysfunction, and chronic pain affecting long-term quality of life (5, 6). The incidence of lymphedema following ALND has been reported to range from 14% to 40% in systematic reviews, representing a substantial burden for survivors (5). Consequently, there has been growing interest in identifying patients who achieve apCR to guide surgical de-escalation toward sentinel lymph node biopsy (SLNB), which carries substantially less morbidity (7). Landmark clinical trials, notably ACOSOG Z1071 (8), SENTINA (9), and SN FNAC (10), demonstrated that SLNB after NAC in initially node-positive patients is technically feasible, though the false-negative rate remains a concern—particularly when fewer than three sentinel nodes are retrieved or when dual-tracer mapping is not employed. The Z1071 trial established a benchmark false-negative rate (FNR) of 12.6%, which decreased to 9.1% when three or more sentinel nodes were resected (8). These findings set the clinical standard against which any predictive model for axillary response must ultimately be measured.

Several clinical and pathological factors have been associated with axillary response following NAC, including molecular subtype, primary tumor size, clinical nodal burden, Ki-67 proliferation index, and clinical response to chemotherapy as assessed by physical examination and imaging (11, 12). However, individual clinical variables have limited predictive accuracy for apCR, and existing clinical models typically achieve areas under the receiver operating characteristic curve (AUC) ranging from 0.60 to 0.70 (13, 14). Kim and colleagues developed a predictive nomogram incorporating tumor stage, nodal status, and receptor profiles, achieving an AUC of 0.72 in external validation but noted substantial heterogeneity across molecular subtypes (13). Tadros and colleagues explored the feasibility of omitting axillary surgery based on breast pCR combined with imaging findings, but acknowledged the limited sensitivity of currently available tools (14). This modest performance underscores the need for complementary predictive biomarkers that capture additional dimensions of tumor biology not reflected in conventional clinical parameters. In parallel, imaging-based assessments including ultrasound, MRI, and positron emission tomography (PET-CT) have been investigated for predicting nodal response, yet their performance as standalone tools remains insufficient for definitive clinical decision-making (15, 16). MRI-based response assessment, while valuable for breast tumor evaluation, has shown sensitivity of only 60–85% for detecting residual axillary disease depending on subtype and assessment criteria (15).

Radiomics, the high-throughput extraction of quantitative features from medical images, has emerged as a promising approach for characterizing tumor heterogeneity and predicting treatment response in breast cancer (17, 18). By quantifying texture patterns, shape properties, and intensity distributions from regions of interest (ROIs) on dynamic contrast-enhanced MRI (DCE-MRI), radiomics may capture phenotypic information complementary to clinical variables (19). The underlying hypothesis is that tumor microenvironment heterogeneity, as reflected in imaging texture features, correlates with chemosensitivity and treatment response through mechanisms related to tumor vascularity, cellularity, and necrosis (17). Several prior studies have explored radiomics-based prediction of breast pathological complete response, demonstrating incremental predictive value when combined with clinical data (20, 21). Braman and colleagues showed that intratumoral and peritumoral radiomic features from pre-treatment DCE-MRI improved prediction of breast pCR, with combined models achieving AUC values exceeding 0.80 in single-center analysis (21). However, most published models focus on breast pCR rather than specifically on axillary pCR, which is arguably the more clinically actionable endpoint for guiding axillary surgical decisions. Furthermore, few studies have simultaneously addressed the rigorous methodological requirements for prediction model development outlined in the Transparent Reporting of a multivariable prediction model for Individual Prognosis Or Diagnosis (TRIPOD) guidelines (22), and the external generalizability of radiomics signatures remains a major challenge, as features may be sensitive to variations in scanner hardware, acquisition protocols, and image reconstruction parameters (23, 24).

Therefore, the aim of this study was to develop and internally validate a combined radiomics-clinical prediction model for axillary pCR in cN+ breast cancer patients after NAC, with the ultimate goal of supporting individualized surgical planning. We hypothesized that integrating DCE-MRI radiomic features with established clinical predictors would improve discrimination beyond clinical variables alone. We report our findings following the TRIPOD guideline for prediction model development studies (Type 2: development and internal validation) (22), incorporating comprehensive calibration assessment, decision curve analysis, and honest acknowledgment of model limitations as recommended by current prediction model reporting standards (25, 26).

## Methods

### Study design and participants

**Figure 1:**
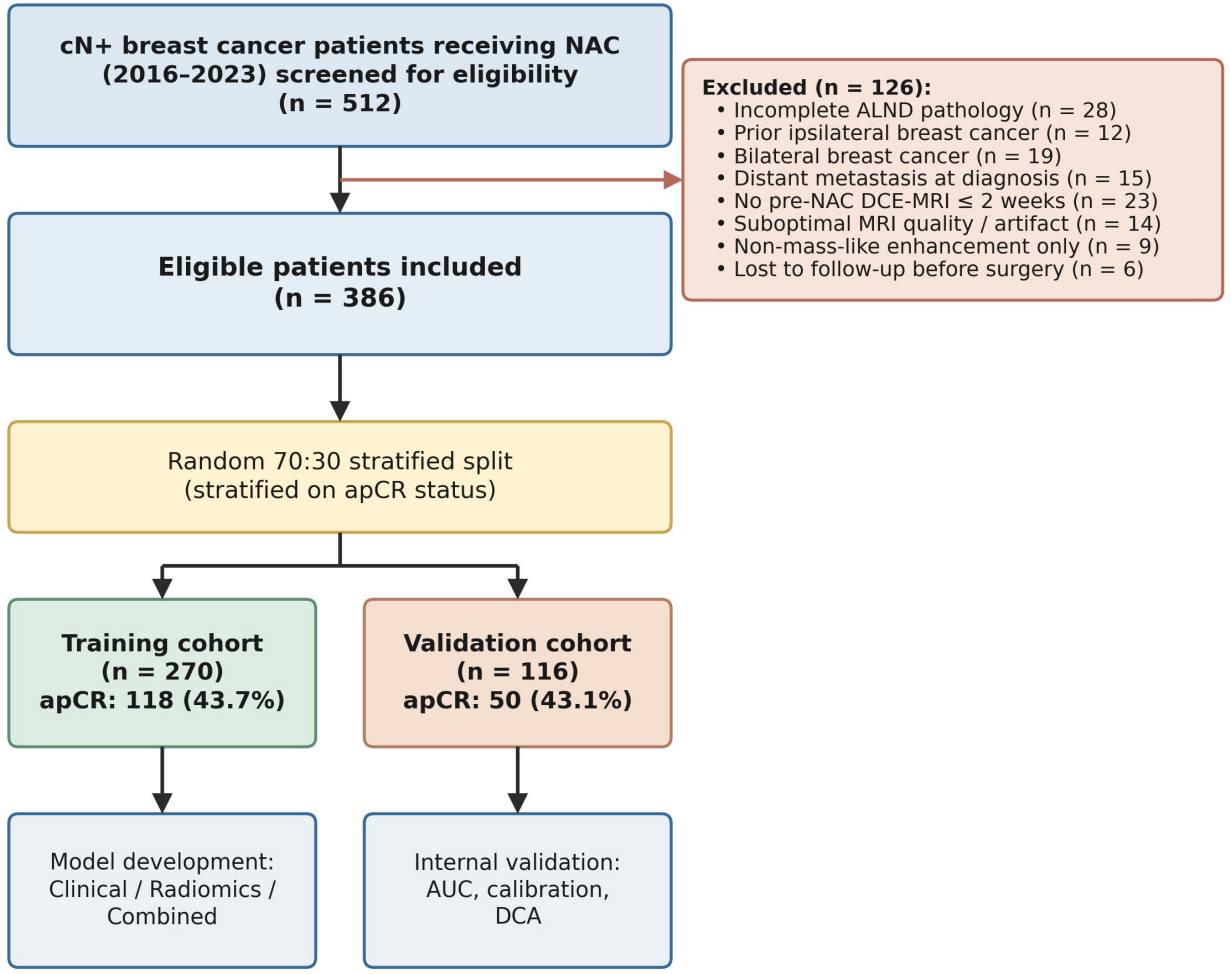
Patient selection flowchart. A total of 512 patients were initially screened, with 126 excluded for predefined criteria, resulting in 386 eligible patients randomly allocated to training (n = 270) and validation (n = 116) cohorts.

This single-center retrospective cohort study was approved by the institutional review board (IRB No. 3256), and the requirement for informed consent was waived due to the retrospective design. We identified 512 consecutive cN+ breast cancer patients who received NAC between January 2016 and December 2023 at The First Hospital of Qinhuangdao. Clinical nodal positivity was defined as biopsy-confirmed axillary lymph node metastasis or imaging-identified suspicious nodes (cortical thickness ≥ 3 mm with loss of fatty hilum on ultrasound or MRI). Exclusion criteria included: incomplete axillary dissection pathology data (n = 28); prior ipsilateral breast cancer (n = 12); bilateral breast cancer (n = 19); distant metastasis at diagnosis (n = 15); absence of pre-NAC DCE-MRI within two weeks of chemotherapy initiation (n = 23); suboptimal MRI quality or motion artifact (n = 14); non-mass-like enhancement only, precluding reliable segmentation (n = 9); and loss to follow-up before surgery (n = 6). After exclusions, 386 patients constituted the final study cohort (**Figure 1**). The overall exclusion rate was 24.6% (126/512); the potential implications of these exclusions for generalizability are addressed in the Discussion section.

### Study endpoint and cohort splitting

The primary endpoint was axillary pathological complete response (apCR), defined as ypN0 (absence of residual invasive or *in situ* carcinoma in all resected axillary lymph nodes) on final ALND pathology. All patients underwent ALND following NAC regardless of clinical response. The 386 eligible patients were randomly split into training (n = 270, 70%) and validation (n = 116, 30%) sets with stratification on apCR status using a fixed random seed to ensure reproducibility.

### Clinical data collection

Clinical and pathological data were extracted from electronic medical records by two investigators independently, with discrepancies resolved by a senior oncologist. Variables collected included: age, menopausal status, tumor size (longest diameter on pre-NAC imaging), clinical T and N stage (AJCC 8th edition), estrogen receptor (ER) status, progesterone receptor (PR) status, HER2 status (immunohistochemistry and/or fluorescence *in situ* hybridization), Ki-67 proliferation index (percentage of positive cells), molecular subtype (Luminal A, Luminal B, HER2-enriched/HR+, HER2-enriched/HR−, and TNBC per the St. Gallen 2013 consensus), and breast clinical complete response (cCR, defined as no palpable residual disease and no residual enhancement on imaging after NAC).

### MRI acquisition and radiomics pipeline

All patients underwent pre-NAC axial DCE-MRI on a 3.0T scanner (Siemens MAGNETOM Skyra or Prisma) using a dedicated breast coil. The standard protocol included a pre-contrast T1-weighted sequence, followed by dynamic contrast-enhanced acquisition after intravenous contrast injection. Imaging parameters were: repetition time 4.5–5.2 ms, echo time 1.7–2.0 ms, flip angle 10°, matrix 384 × 384, slice thickness 1.0–1.5 mm, and temporal resolution approximately 60 seconds per phase. Images were acquired at six time points (one pre-contrast and five post-contrast). The gadolinium-based contrast agent was gadoteric acid (Dotarem), administered at a dose of 0.1 mmol/kg body weight at an injection rate of 2.0 mL/s using a power injector, immediately followed by a 20 mL saline flush delivered at the same rate. A transverse fat-suppressed three-dimensional T1-weighted gradient-echo volumetric sequence (VIBE) was employed, with the following additional parameters: field of view 320–340 mm, receiver bandwidth 380–440 Hz/pixel, parallel imaging (GRAPPA) acceleration factor 2, and frequency-selective fat suppression. A total of six dynamic phases were acquired (one pre-contrast and five consecutive post-contrast phases) at a temporal resolution of approximately 60 seconds per phase, spanning approximately six minutes after contrast injection. Images were reconstructed online using the vendor standard GRAPPA reconstruction, and no additional smoothing or post-processing filter was applied prior to radiomic feature extraction. A complete itemized list of DCE-MRI acquisition and reconstruction parameters is provided in **Supplementary Table 5**.

Region of interest (ROI) segmentation of the primary breast tumor was performed on the first post-contrast subtraction images using ITK-SNAP (version 3.8) by two breast radiologists (Reader A with 8 years and Reader B with 12 years of experience) who were blinded to pathological outcomes. Segmentation was performed independently, followed by consensus review. Intraclass correlation coefficients (ICC) were calculated for all extracted features between the two readers; features with ICC < 0.80 were excluded from further analysis. Segmentation was performed slice by slice across the entire tumor to generate a three-dimensional volume of interest rather than a single representative slice. Readers were instructed to encompass the whole enhancing tumor while excluding visible intratumoral vessels, macroscopic necrosis, and adjacent normal fibroglandular parenchyma; an inner margin of approximately 1–2 mm from the enhancing tumor border was maintained to minimize partial-volume contamination, and for multifocal disease the largest index lesion was segmented. The ICC was computed using a two-way random-effects model with absolute agreement. A total of 1,316 radiomic features were extracted using PyRadiomics (version 3.0.1) (27), encompassing first-order statistics, shape features, gray-level co-occurrence matrix (GLCM), gray-level run-length matrix (GLRLM), gray-level size zone matrix (GLSZM), gray-level dependence matrix (GLDM), and neighboring gray-tone difference matrix (NGTDM) features. Wavelet and Laplacian of Gaussian (LoG) filters with sigma values of 2, 3, and 5 mm were applied prior to feature extraction. Images were resampled to isotropic 1 × 1 × 1 mm voxels using B-spline interpolation, and a fixed bin width of 25 arbitrary intensity units was applied for intensity discretization, as MRI signal intensities lack a standardized physical unit analogous to Hounsfield units in computed tomography. After ICC filtering (1,316 → 412 features retained with ICC ≥ 0.80), features with pairwise absolute Spearman correlation |ρ| ≥ 0.90 were further removed (retaining the feature with the higher ICC), reducing the candidate pool from 412 to 18 features for LASSO selection.

### Feature selection and rad-score construction

**Figure 2:**
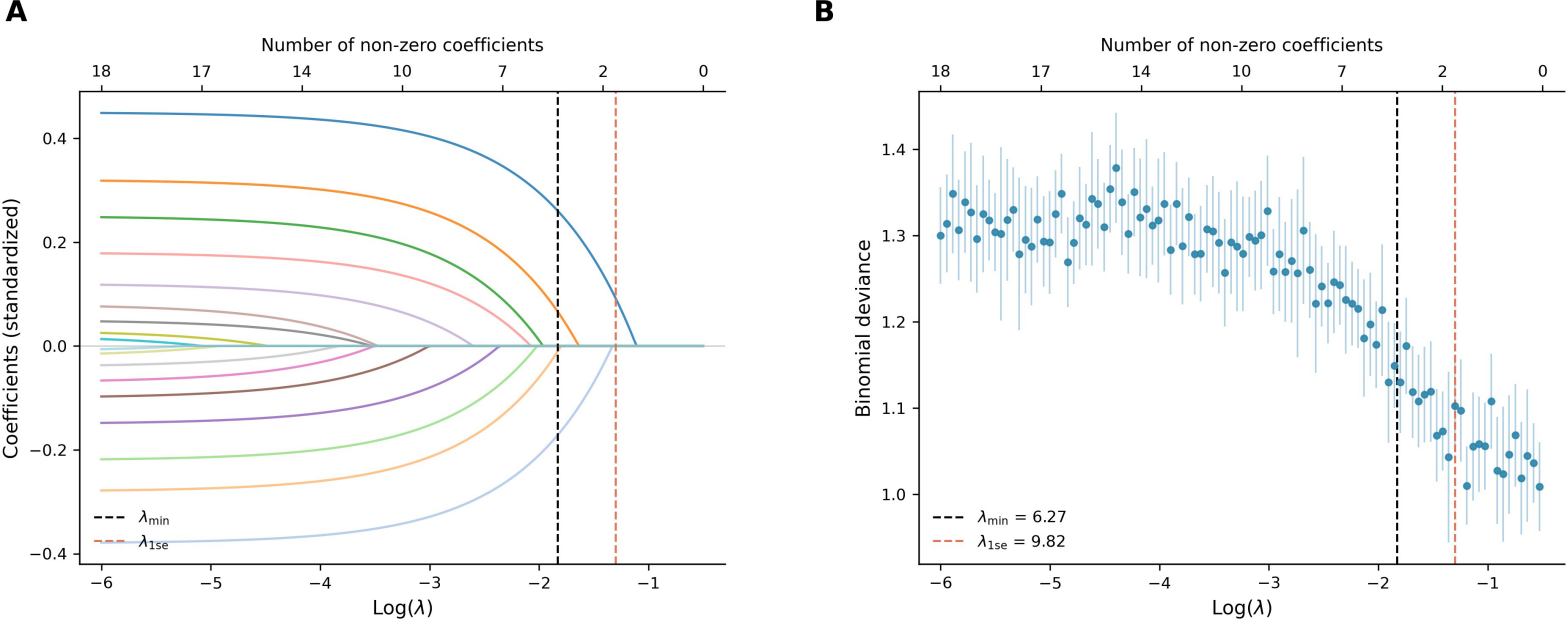
LASSO feature selection. **(A)** Coefficient profile plot showing the regularization paths of all 18 candidate radiomic features as a function of Log(λ). Vertical dashed lines mark λmin and λ1se. **(B)** Ten-fold cross-validation curve of binomial deviance versus Log(λ); λmin retained eight features.

The 18 candidate radiomic features were subjected to least absolute shrinkage and selection operator (LASSO) logistic regression with 10-fold cross-validation to identify the optimal regularization parameter (λ). We selected λmin (the λ value minimizing mean cross-validated deviance) as the primary model, and also evaluated λ1se (one standard error rule) as a more parsimonious alternative that retained 7 features. At λmin, eight features were retained with non-zero coefficients (**Figure 2**). Notably, one feature (Wavelet-HLH_GLCM_Imc1) had a coefficient of only −0.017, contributing negligibly to the Rad-score. A sensitivity analysis removing this feature demonstrated no loss in AUC (ΔAUC < 0.001); however, it was retained for consistency with the λmin solution. The Rad-score was calculated as the linear combination of the eight selected features weighted by their LASSO coefficients. The complete formula and intercept are provided in **Supplementary Table 1**.

**Figure 3:**
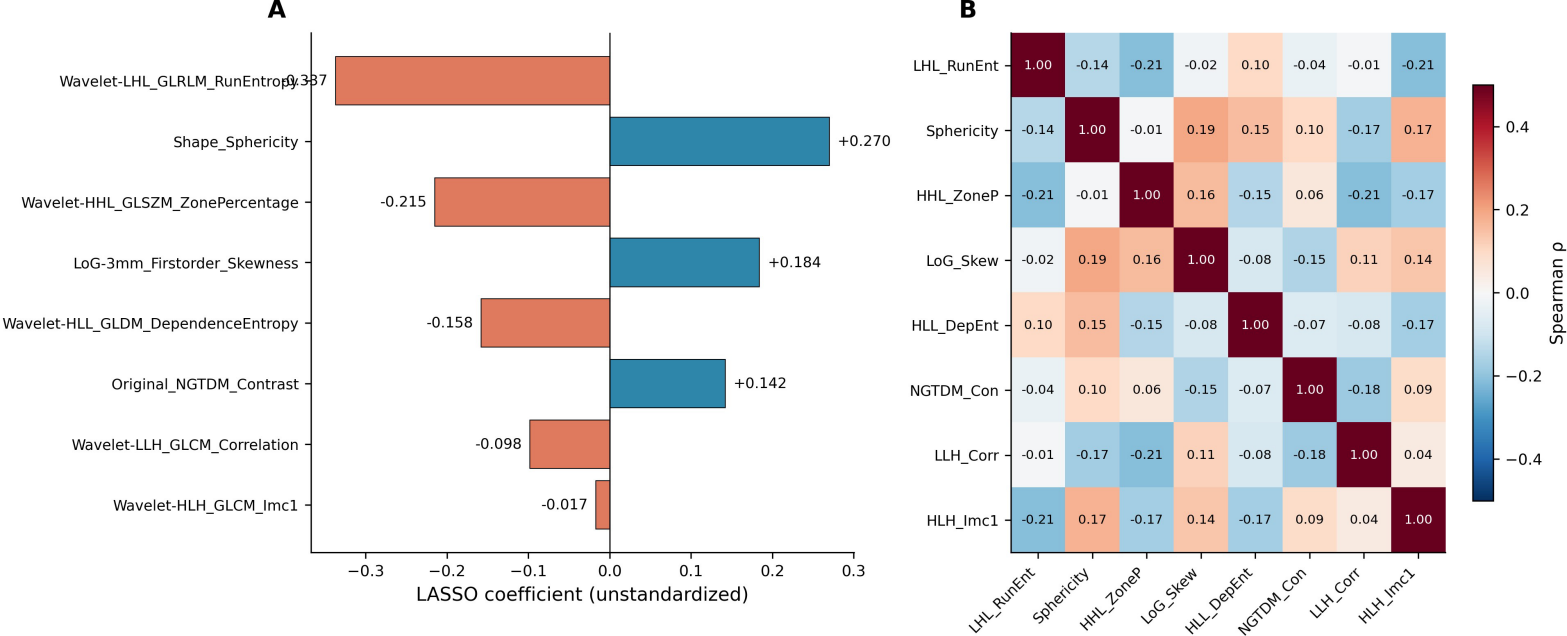
Selected radiomic features. **(A)** LASSO coefficients of the eight retained features ranked by absolute magnitude, displayed on the unstandardized (original feature) scale. **(B)** Spearman correlation matrix confirming weak inter-feature correlations (all |ρ| < 0.25).

**Figure 3** shows the LASSO coefficients for the eight retained features on the unstandardized (original feature) scale, along with their pairwise Spearman correlation matrix. Note that **Figure 2A** displays coefficients on the standardized scale (features normalized to zero mean and unit variance prior to penalization), whereas **Figure 3A** reports coefficients on the original feature scale as used in the Rad-score formula. All pairwise correlations among selected features were weak (|ρ| < 0.25), confirming minimal multicollinearity. The strongest contributor was Wavelet-LHL_GLRLM_RunEntropy (coefficient = −0.337), a texture feature capturing run-length heterogeneity in the LHL wavelet decomposition. Shape_Sphericity (coefficient = +0.270) quantifies the degree to which the tumor approximates a sphere, with higher sphericity associated with increased apCR probability.

As a sensitivity analysis, the λ1se model retaining 7 features achieved a training AUC of 0.718 (95% CI, 0.658–0.778) and validation AUC of 0.691 (95% CI, 0.596–0.782). The modest reduction in validation AUC from 0.703 to 0.691 suggests that the additional feature in the λmin model provided marginal discriminatory benefit, consistent with the concern that λmin may retain features with weak signal. Complete performance metrics for the λ1se model are provided in **Supplementary Table 4**.

### Clinical predictor selection

Candidate clinical predictors were first screened by univariate logistic regression in the training set. Variables with P < 0.10 were entered into a multivariate logistic regression model. Variables retaining P < 0.10 in the multivariate model were included in the final clinical model. The multivariate selection was performed using backward elimination with the likelihood ratio test. All odds ratios (ORs) are reported with 95% confidence intervals (CIs) derived from the profile likelihood method. Molecular subtype was additionally evaluated as a four-level categorical variable (Luminal, HER2+/HR+, HER2+/HR−, TNBC) in a supplementary analysis, and subtype-stratified model performance is reported.

### Model construction and evaluation

Three prediction models were constructed in the training set: (1) a clinical model incorporating the selected clinical predictors; (2) a radiomics model using the Rad-score alone; and (3) a combined model incorporating both clinical predictors and the Rad-score. Model discrimination was assessed by AUC with 95% CIs computed using 2,000-iteration stratified bootstrap resampling. Pairwise AUC comparisons between models were performed using the bootstrap method (28). Training-set AUC estimates are subject to optimistic bias, as LASSO feature selection and model fitting were performed on the same data without nested cross-validation. Therefore, interpretation of the training AUC should be cautious, and we regard the validation AUC as the primary performance metric.

Sensitivity, specificity, accuracy, positive predictive value (PPV), and negative predictive value (NPV) were computed at a classification threshold of 0.42, which corresponds to the Youden index-optimized cutoff derived exclusively from the training set and applied unchanged to the validation set. To assess threshold robustness, we additionally report model performance across a range of thresholds (0.30–0.55) and at the equal-error-rate threshold. Calibration was assessed by the Hosmer-Lemeshow goodness-of-fit test (8 groups), calibration-in-the-large (intercept), and calibration slope (25). We note that the training-set calibration slope is 1.000 by construction for logistic regression models evaluated on their own training data; this value therefore has no diagnostic meaning and should not be interpreted as evidence of good calibration. Overall prediction accuracy was quantified by the Brier score and Brier Skill Score (BSS), defined as improvement over baseline prevalence-based prediction (i.e., the reference model predicts prevalence = 0.435 for all patients). Decision curve analysis (DCA) was performed to evaluate clinical utility across a range of threshold probabilities (29). To quantify and correct for optimism in the apparent (training-set) discrimination, we additionally computed bias-corrected AUCs using the Harrell enhanced (.632+) bootstrap with 1,000 resamples, in which the optimism (apparent performance minus out-of-bag performance) was estimated and subtracted from the apparent AUC. Both the apparent and the optimism-corrected training AUCs are reported in **Table 1**.

**Table 1:** Discrimination and calibration metrics of the three prediction models.

Model	Cohort	AUC (95% CI)	Optimism-corrected AUC	Sen	Spe	Acc	PPV	NPV	Brier	Cal. Slope	BSS
Clinical	Train	0.667 (0.601–0.733)	0.651	0.695	0.605	0.644	0.577	0.719	0.226	1.000*	0.080
Clinical	Val	0.683 (0.585–0.785)	—	0.640	0.636	0.638	0.571	0.700	0.220	0.903	0.102
Radiomics	Train	0.735 (0.678–0.795)	0.661	0.627	0.730	0.685	0.643	0.716	0.205	1.000*	0.166
Radiomics	Val	0.608 (0.497–0.710)	—	0.500	0.682	0.603	0.543	0.643	0.243	0.649	0.008
Combined	Train	0.785 (0.728–0.835)	0.742	0.754	0.711	0.730	0.669	0.788	0.186	1.000*	0.242
Combined	Val	0.703 (0.610–0.792)	—	0.640	0.606	0.621	0.552	0.690	0.217	0.811	0.116

*Training-set calibration slope = 1.000 by construction for logistic regression. Threshold 0.42 (Youden, training) applied unchanged to validation. BSS reference: prevalence-based prediction (base rate 0.435). Optimism = apparent training AUC minus optimism-corrected AUC (≈0.016 clinical, 0.074 radiomics, 0.043 combined). AUC, area under the ROC curve; Sen, sensitivity; Spe, specificity; Acc, accuracy; PPV/NPV, positive/negative predictive value; Cal. Slope, calibration slope; BSS, Brier Skill Score.

Optimism-corrected training AUCs (enhanced.632+ bootstrap, 1,000 resamples) are reported in the added column.

Risk stratification was performed using training-set-derived tertile cutoffs applied to both cohorts. All analyses were conducted in Python 3.10 (scikit-learn 1.3, statsmodels 0.14) and R 4.3 (glmnet, rms, dcurves packages). Statistical significance was set at two-sided P < 0.05.

### Sample size considerations

We conducted a *post hoc* sample size assessment following the framework of Riley and colleagues (30). The training set contained 118 events (apCR) and 152 non-events across 5 final predictor variables (Rad-score counted as one composite variable), yielding an events per variable (EPV) ratio of 23.6. When considering the 18 candidate features evaluated during LASSO selection, the effective EPV was 6.6, below the commonly recommended minimum of 10 (30). We applied the pmsampsize approach (R package pmsampsize) to estimate the minimum required sample size based on the anticipated model performance. Using a Cox-Snell R² of 0.15 (estimated from the training data), the minimum sample size was 298 with at least 130 events, suggesting our training set (n = 270, 118 events) was marginally adequate for final model development but undersized for the candidate screening phase. These results support the need for external validation in a larger multicenter cohort. To mitigate the instability associated with an EPV below 10 during the candidate-screening phase, feature dimensionality was aggressively reduced before modeling (ICC filtering followed by correlation pruning to 18 candidates), and LASSO L1 penalization was used specifically because its shrinkage property stabilizes coefficient estimation when events are limited. The stability of the eight selected features was further examined by repeating the LASSO selection across 1,000 bootstrap resamples of the training set; each of the eight features retained in the primary model was selected in more than 70% of resamples, supporting the reproducibility of the signature (bootstrap selection frequencies provided in **Supplementary Table 6**). We nonetheless regard the candidate-screening EPV of 6.6 as a genuine limitation and emphasize that the final reported performance should be interpreted in light of it.

### TRIPOD and radiomics quality reporting

This study was reported following the TRIPOD guidelines for prediction model development and internal validation (Type 2) (22). A completed TRIPOD checklist is provided as **Supplementary Table 2**. The Radiomics Quality Score (RQS) self-assessment is provided as **Supplementary Table 3**. We acknowledge that this is a single-center study without external validation and that no phantom stability testing or ComBat harmonization was performed, both of which limit the RQS and the generalizability of the radiomic features across different scanner platforms and acquisition protocols.

## Results

### Baseline characteristics

A total of 386 patients were included, with 168 (43.5%) achieving apCR. Baseline characteristics of the training and validation cohorts are summarized in **Table 2**. The two cohorts were well balanced across all variables (all P > 0.05), except for cN stage, which showed a marginally significant difference (P = 0.045). The mean age was 52.0 ± 10.7 years, with 59.8% of patients being postmenopausal. The mean pre-NAC tumor size was 39.1 ± 17.9 mm. The distribution of molecular subtypes was as follows: Luminal A 7.0%, Luminal B 44.6%, HER2-enriched/HR+ 21.5%, HER2-enriched/HR− 6.5%, and TNBC 20.5%. Notably, the proportion of TNBC cases was 22.6% in the training set versus 15.5% in the validation set, a distributional difference that may have influenced model transportability, as discussed further below.

**Table 2:** Baseline characteristics of the training and validation cohorts.

Variable	Training (n=270)	Validation (n=116)	Total (n=386)	P value
Age, years (mean ± SD)	51.8 ± 10.9	52.6 ± 10.4	52.0 ± 10.7	0.503
Postmenopausal, n (%)	160 (59.3)	71 (61.2)	231 (59.8)	0.719
Tumor size, mm (mean ± SD)	39.4 ± 18.2	38.5 ± 17.3	39.1 ± 17.9	0.653
cT stage, n (%)				0.426
T1	35 (13.0)	18 (15.5)	53 (13.7)	
T2	143 (53.0)	65 (56.0)	208 (53.9)	
T3	66 (24.4)	23 (19.8)	89 (23.1)	
T4	26 (9.6)	10 (8.6)	36 (9.3)	
cN stage, n (%)				0.045
N1	184 (68.1)	87 (75.0)	271 (70.2)	
N2	63 (23.3)	21 (18.1)	84 (21.8)	
N3	23 (8.5)	8 (6.9)	31 (8.0)	
ER-positive, n (%)	193 (71.5)	81 (69.8)	274 (71.0)	0.741
PR-positive, n (%)	156 (57.8)	68 (58.6)	224 (58.0)	0.874
HER2-positive, n (%)	76 (28.1)	32 (27.6)	108 (28.0)	0.905
Ki-67, % (mean ± SD)	38.2 ± 20.1	37.5 ± 19.8	38.0 ± 20.0	0.753
Molecular subtype, n (%)				0.328
Luminal A	19 (7.0)	8 (6.9)	27 (7.0)	
Luminal B	114 (42.2)	58 (50.0)	172 (44.6)	
HER2+/HR+	57 (21.1)	26 (22.4)	83 (21.5)	
HER2+/HR−	19 (7.0)	6 (5.2)	25 (6.5)	
TNBC	61 (22.6)	18 (15.5)	79 (20.5)	
Breast clinical CR, n (%)	98 (36.3)	40 (34.5)	138 (35.8)	0.735
apCR (ypN0), n (%)	118 (43.7)	50 (43.1)	168 (43.5)	0.912

SD, standard deviation; cT, clinical tumor stage; cN, clinical nodal stage; ER, estrogen receptor; PR, progesterone receptor; HER2, human epidermal growth factor receptor 2; TNBC, triple-negative breast cancer; CR, complete response; apCR, axillary pathological complete response.

### Molecular subtype and apCR rate

The apCR rate varied substantially across molecular subtypes in the training set: Luminal A 26.3% (5/19), Luminal B 36.0% (41/114), HER2+/HR+ 57.9% (33/57), HER2+/HR− 52.6% (10/19), and TNBC 47.5% (29/61). In univariate analysis, the Luminal versus non-Luminal comparison yielded OR = 0.48 (95% CI, 0.29–0.78; P = 0.003), which was a stronger predictor than HER2 status alone (OR = 2.07; P = 0.008). When molecular subtype was entered as a four-level categorical variable in the multivariate model, it did not achieve independent significance (likelihood ratio test P = 0.12) after adjustment for HER2 status, Ki-67, tumor size, and breast cCR. The HER2 binary variable was therefore retained in the final model for parsimony, though we acknowledge that the study was underpowered for subtype-level analysis and that future multicenter studies should formally evaluate subtype-specific prediction. To directly test whether molecular subtype provides independent predictive information beyond the combined model, we additionally fitted an extended model adding the four-level subtype variable to the five combined-model predictors. Subtype did not reach significance in this extended model (likelihood ratio test P = 0.18), the validation AUC was essentially unchanged (0.703 versus 0.706, DeLong P = 0.79), and the variance inflation factors for HER2 status and Ki-67 increased above 2.5, indicating collinearity between subtype and the receptor-based predictors. Molecular subtype was therefore not retained as an independent predictor, and HER2 status was kept as a parsimonious biological surrogate (extended-model results in **Supplementary Table 7**).

### Rad-score distribution

**Figure 4:**
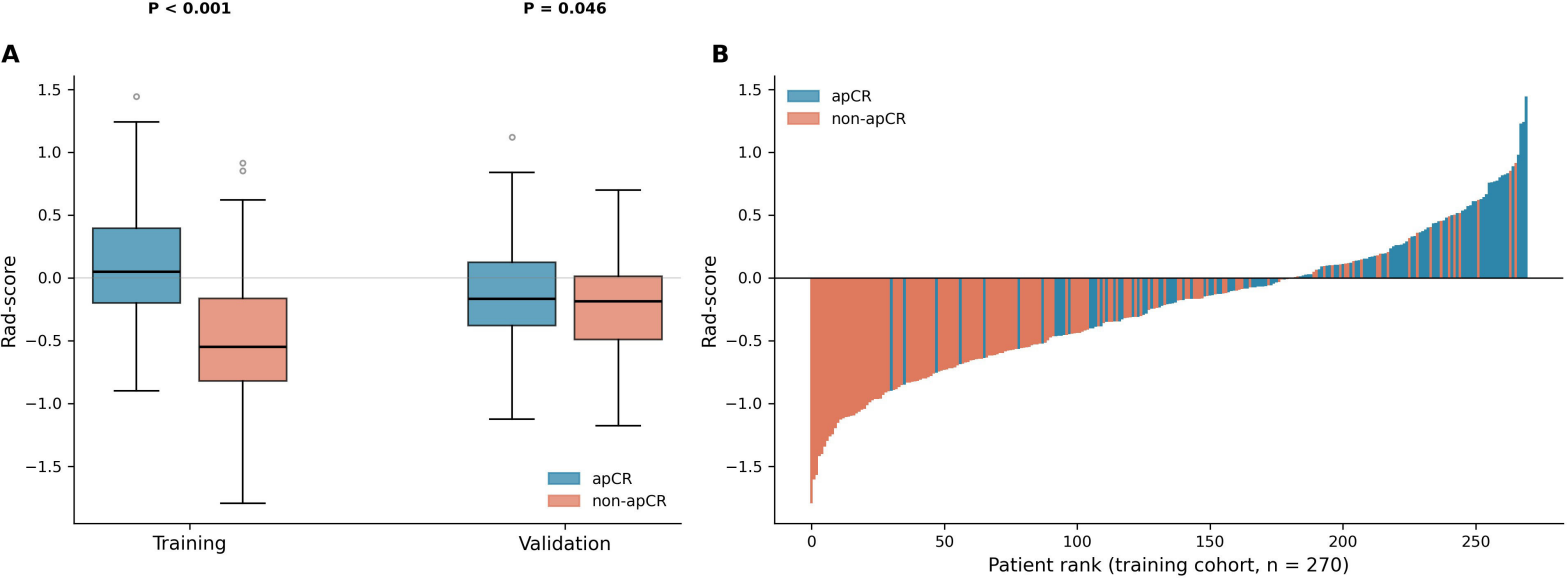
Rad-score distribution by apCR status. **(A)** Box plots comparing Rad-scores between apCR (blue) and non-apCR (orange) groups in the training and validation cohorts. Central lines represent medians; boxes represent interquartile ranges. P values from Mann-Whitney U test. **(B)** Waterfall plot of individual Rad-scores in the training cohort ranked by magnitude, colored by apCR status.

The Rad-score was significantly higher in patients who achieved apCR compared with those who did not in both cohorts: training set median 0.08 (IQR, −0.31 to 0.42) versus −0.53 (IQR, −0.92 to −0.10), Mann-Whitney U = 13,188, P < 0.001; validation set median −0.05 (IQR, −0.35 to 0.15) versus −0.30 (IQR, −0.60 to 0.06), U = 2,008, P = 0.046 (**Figure 4A**). The effect size was considerably smaller in the validation set, consistent with the expected shrinkage of a training-set-optimized signature. The waterfall plot (**Figure 4B**) illustrates the distribution of Rad-scores in the training cohort ranked by magnitude, with substantial overlap between apCR and non-apCR groups in the mid-range, reflecting the moderate discriminatory power of the radiomics signature.

### Clinical predictor selection

Univariate and multivariate logistic regression results are presented in **Tables 3**, **4**. In univariate analysis, four variables achieved P < 0.10: tumor size (OR = 0.98 per mm, 95% CI 0.97–1.00, P = 0.024), HER2-positive status (OR = 2.07, 95% CI 1.21–3.54, P = 0.008), Ki-67 (OR = 1.02 per %, 95% CI 1.00–1.03, P = 0.039), and breast clinical CR (OR = 2.28, 95% CI 1.37–3.80, P = 0.002). All four variables retained P < 0.10 in the multivariate model and were included in the final clinical prediction model.

**Table 3:** Univariate logistic regression analysis of clinical predictors for apCR (training set).

Variable	OR	95% CI	P value
Age, per year	1.00	0.98–1.02	0.983
Postmenopausal	0.85	0.53–1.37	0.504
Tumor size, per mm	0.98	0.97–1.00	0.024
cT3–4 vs T1–2	0.79	0.48–1.30	0.354
cN2–3 vs N1	0.72	0.43–1.21	0.213
ER-positive	0.67	0.40–1.12	0.127
PR-positive	0.76	0.47–1.22	0.252
HER2-positive	2.07	1.21–3.54	0.008
Ki-67, per %	1.02	1.00–1.03	0.039
Breast clinical CR	2.28	1.37–3.80	0.002

OR, odds ratio; CI, confidence interval; other abbreviations as in **Table 2**.

**Table 4:** Multivariate logistic regression analysis of clinical predictors for apCR (training set).

Variable	Adjusted OR	95% CI	P value
Tumor size, per mm	0.98	0.97–1.00	0.024
HER2-positive	1.69	0.96–2.97	0.068
Ki-67, per %	1.01	1.00–1.03	0.058
Breast clinical CR	2.00	1.18–3.41	0.011

All four variables with P < 0.10 were retained in the final clinical model. Abbreviations as in **Tables 2**, **3**. The OR for Rad-score is reported per 1 standard deviation (SD = 0.55) increase. The corresponding per-unit OR is 16.9.

### Model performance

**Figure 5:**
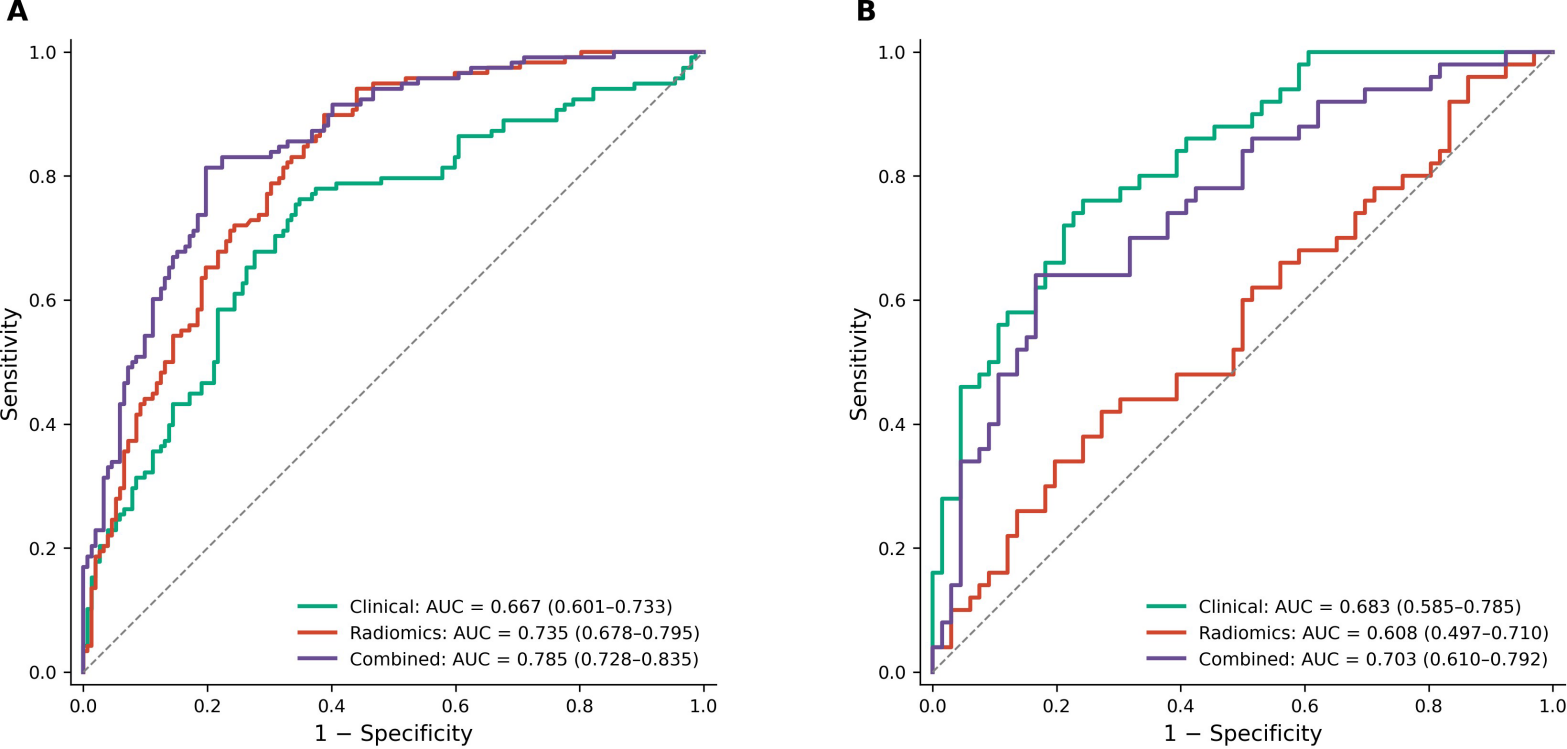
Receiver operating characteristic (ROC) curves. **(A)** Training cohort (n = 270). **(B)** Validation cohort (n = 116). The 95% confidence intervals in parentheses were derived from 2,000-iteration stratified bootstrap resampling, consistent with **Table 1**. The combined model (purple) achieved the highest AUC in both cohorts, though the difference versus the clinical model was not statistically significant in validation (ΔAUC = 0.020, P = 0.713).

The performance metrics of the three models are summarized in **Table 1**. In the training set, the combined model achieved the highest AUC of 0.785 (95% CI, 0.728–0.835), followed by the radiomics model (AUC = 0.735, 95% CI 0.678–0.795) and the clinical model (AUC = 0.667, 95% CI 0.601–0.733). In the validation set, the combined model achieved AUC = 0.703 (95% CI, 0.610–0.792), compared with the clinical model (AUC = 0.683, 95% CI 0.585–0.785) and the radiomics model (AUC = 0.608, 95% CI 0.497–0.710). The ROC curves for both cohorts are shown in **Figure 5**, with 95% CIs derived from the same 2,000-iteration bootstrap procedure used in **Table 1**. After bias correction with the enhanced (.632+) bootstrap, the optimism-corrected training AUCs were 0.742 for the combined model, 0.661 for the radiomics-only model, and 0.651 for the clinical-only model, corresponding to optimism of approximately 0.043, 0.074, and 0.016, respectively. The substantially larger optimism of the radiomics-only model is consistent with its pronounced training-to-validation AUC decline and indicates that the radiomic component was the principal source of overfitting.

Pairwise AUC comparisons are presented in **Table 5**.

**Table 5:** Pairwise AUC comparisons (bootstrap, 2,000 iterations).

Comparison	Training ΔAUC	P	Validation ΔAUC	P
Combined vs Clinical	+0.118	<0.001	+0.020	0.713
Combined vs Radiomics	+0.050	0.008	+0.094	0.004
Radiomics vs Clinical	+0.068	0.130	−0.075	0.347

Pairwise AUC comparisons used bootstrap resampling with 2,000 iterations. ΔAUC, difference between models in each cohort.

### Calibration and clinical utility

**Figure 6:**
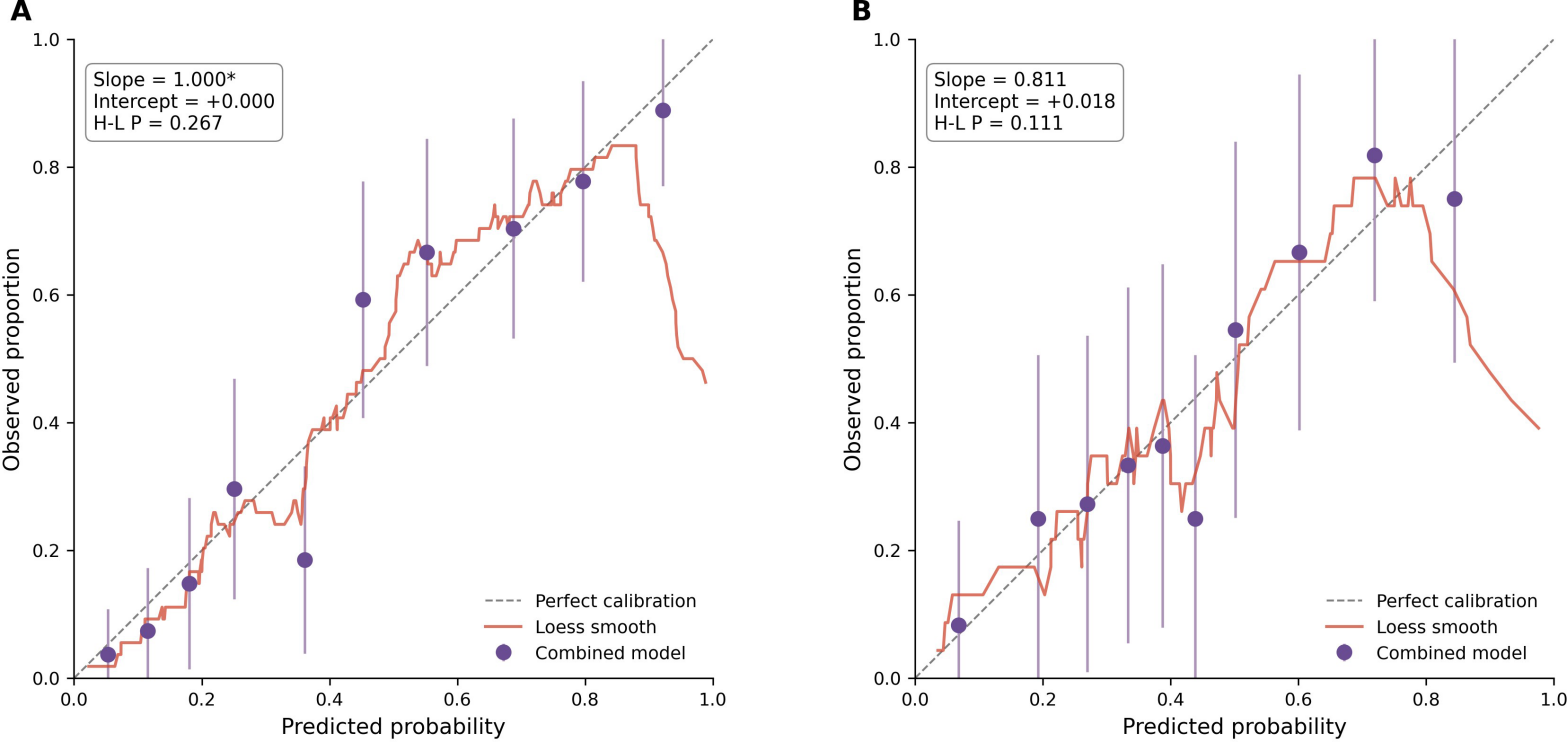
Calibration plots. **(A)** Training cohort (n = 270). The calibration slope of 1.000 is a mathematical property of logistic regression assessed on its own training data (slope = 1 by construction). **(B)** Validation cohort (n = 116). Points represent decile-grouped observed versus predicted probabilities for the combined model, with 95% confidence intervals. Loess smoothing with conservative span (≥ 0.75) was applied. Hosmer-Lemeshow test results and calibration-in-the-large intercept are shown.

Calibration plots and Hosmer-Lemeshow test results are shown in **Figure 6**. The Hosmer-Lemeshow test was non-significant for the combined model in both cohorts (training: χ² = 9.97, df = 8, P = 0.267; validation: χ² = 13.04, df = 8, P = 0.111), indicating no statistically significant deviation from perfect calibration. The training-set calibration slope of 1.000 is a mathematical property of logistic regression (slope = 1 by construction when assessed on training data) and does not provide evidence regarding calibration quality. The validation-set calibration slope of 0.811 indicates moderate overfitting, meaning the model was over-confident in its predictions at the extremes of the probability range. The calibration intercept in validation was 0.018, close to the ideal of 0, suggesting adequate calibration-in-the-large (i.e., the mean predicted probability closely matched the observed event rate). The Brier Skill Score for the combined model in validation was 0.116, indicating an 11.6% improvement over predicting the base rate for all patients. Because training-set calibration and Brier metrics are biased by overfitting, we emphasize the validation-set BSS of 0.116 as the primary accuracy measure. Validation-set decile estimates should be interpreted cautiously, as each decile contains approximately 12 patients, limiting precision; loess smoothing in the calibration plot was therefore performed with a conservative span (≥ 0.75) to avoid over-interpretation of fluctuations.

**Figure 7:**
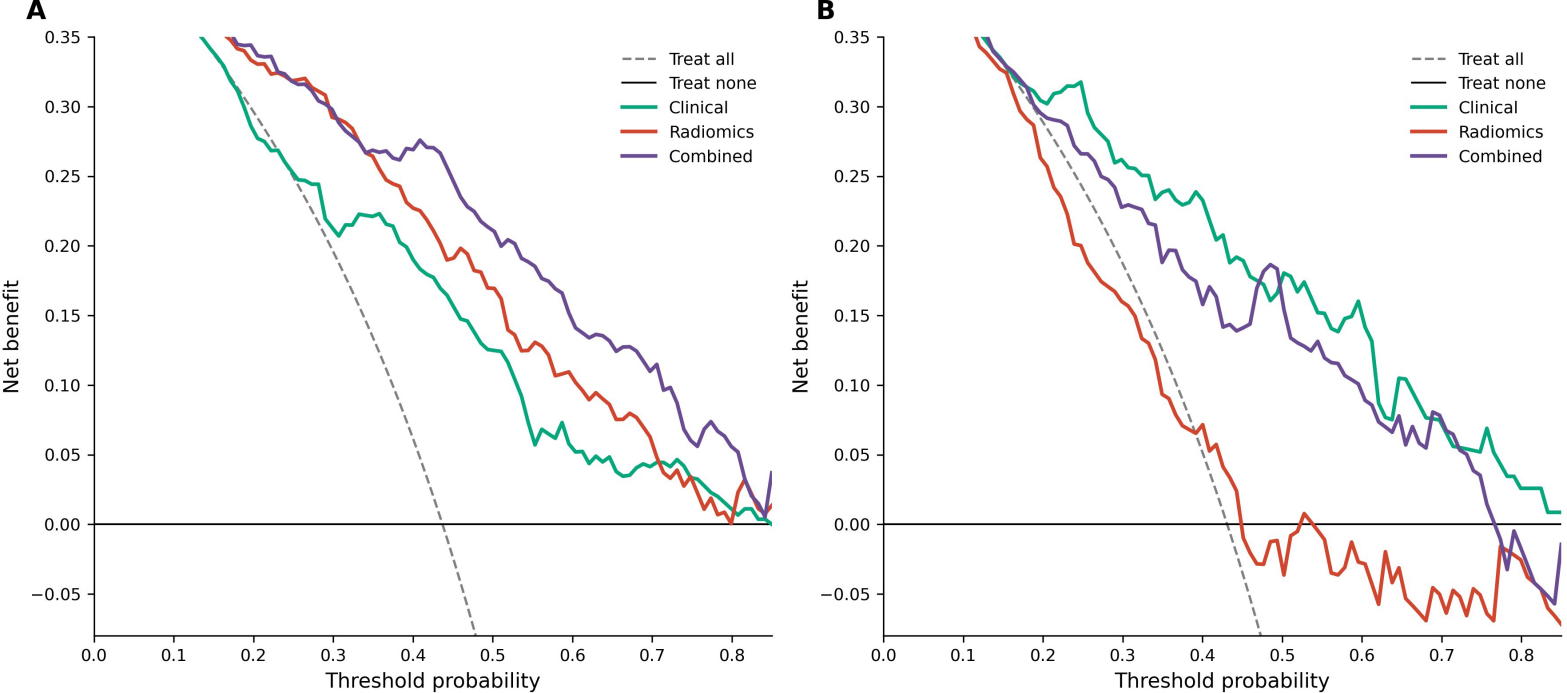
Decision curve analysis. **(A)** Training cohort. **(B)** Validation cohort. The combined model (purple) shows positive net benefit across threshold probabilities of approximately 10–45% in training and 20–45% in validation. Beyond approximately 0.45, all model curves converge to zero or negative net benefit.

Decision curve analysis is presented in **Figure 7**. In the training set, the combined model provided the highest net benefit across threshold probabilities of approximately 10–45%, beyond which all model curves approached zero net benefit. In the validation set, the combined model maintained positive net benefit across the clinically relevant threshold range of approximately 20–45%, exceeding the “treat all” and “treat none” strategies. The clinical model showed comparable or slightly superior net benefit in the validation set at thresholds approaching 0.40, consistent with the non-significant AUC difference between the two models in this cohort. At a representative threshold of 0.30, the net benefit difference between the combined and clinical models in the validation set was approximately 0.02, indicating minimal clinical utility gain from adding the Rad-score.

### Nomogram

**Figure 8:**
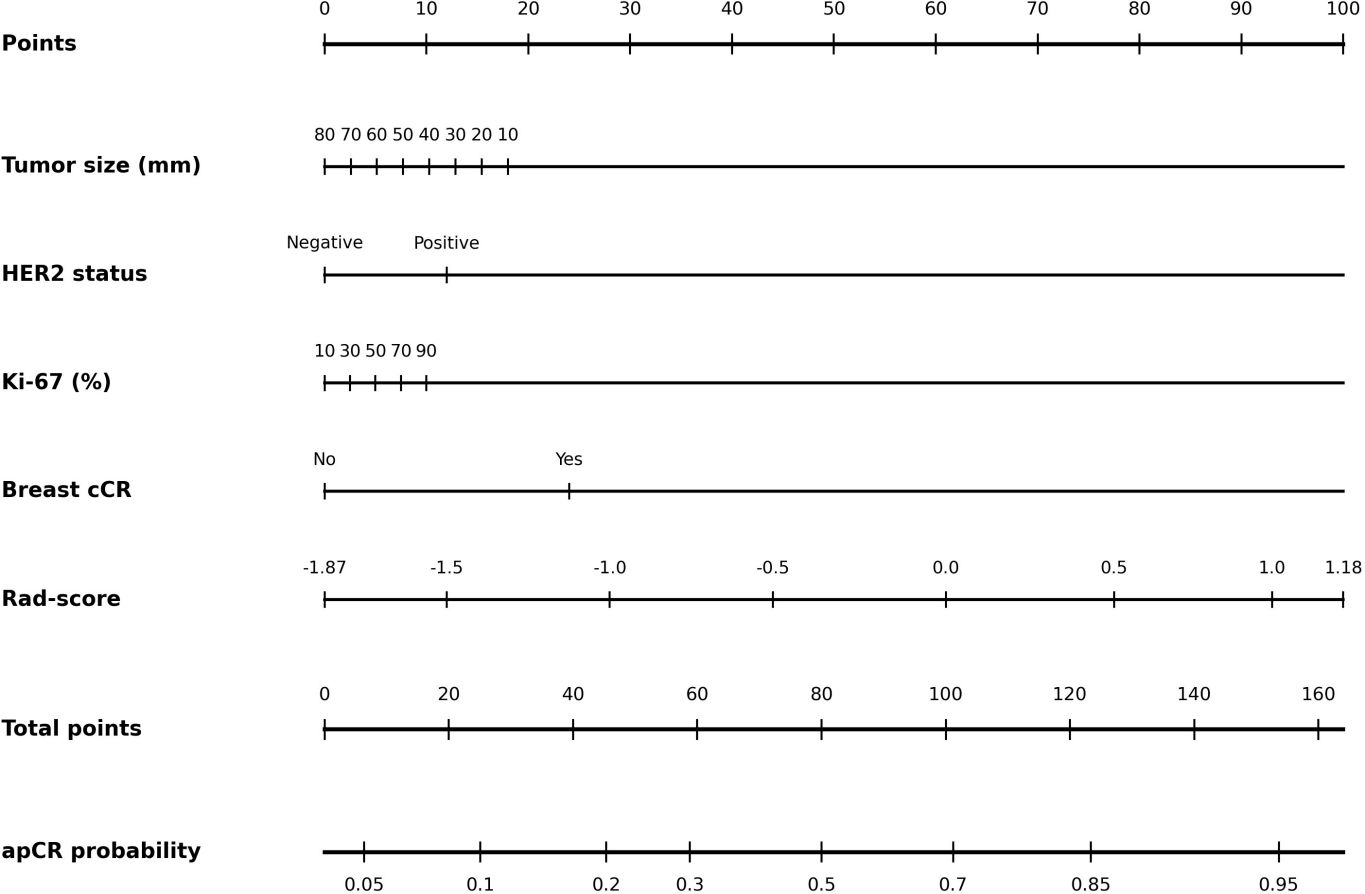
Nomogram for predicting axillary pathological complete response (apCR) after neoadjuvant chemotherapy. Each predictor value corresponds to a point score (top axis); total points are converted to predicted apCR probability (bottom axis). The Rad-score contributes 100 of 164 maximum total points (~61%). The Rad-score axis is restricted to the observed data range.

A nomogram incorporating the five predictors of the combined model was constructed for bedside use (**Figure 8**). The maximum possible point allocations were: Rad-score 100 points, breast cCR 24 points, tumor size 18 points, Ki-67–10 points, and HER2 status 12 points, yielding a maximum total of 164 points. The Rad-score contributed approximately 61% of the total point allocation (100/164), with the four clinical variables collectively contributing the remaining 39%. The Rad-score axis was truncated to the observed 1st–99th percentile range (−1.87 to +1.18) to prevent extrapolation beyond the training data range.

### Combined model multivariable analysis

**Figure 9:**
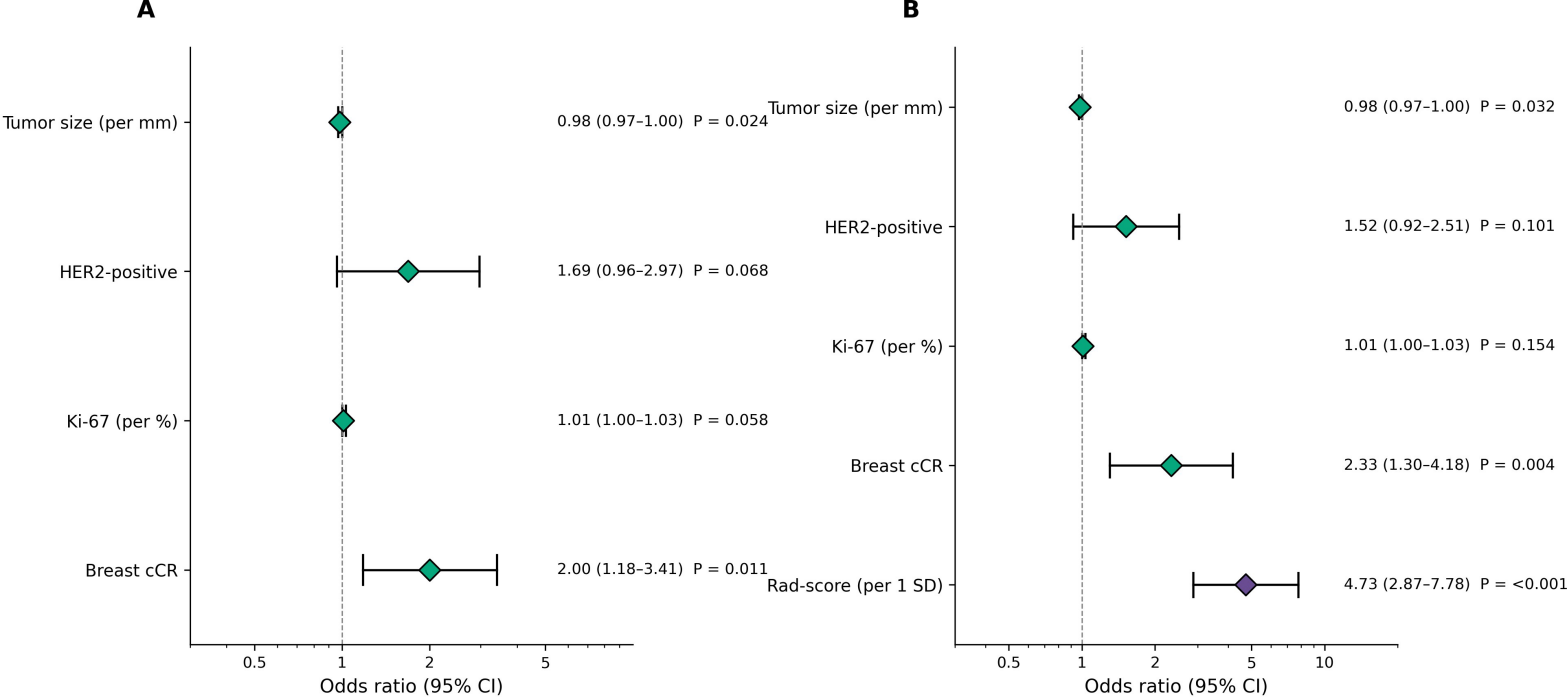
Forest plot of odds ratios. **(A)** Clinical multivariable model. **(B)** Combined model (clinical + Rad-score). The Rad-score OR is reported per 1 SD increase (SD = 0.55). The per-unit OR = exp(ln(4.73)/0.55) = 16.9. Error bars represent 95% confidence intervals. Diamond markers indicate point estimates.

**Figure 9** presents the forest plots for the clinical-only and combined multivariable models. In the combined model, breast clinical CR (adjusted OR = 2.33, 95% CI 1.30–4.18, P = 0.004) and Rad-score (adjusted OR = 4.73 per 1 SD, 95% CI 2.87–7.78, P < 0.001) were the strongest independent predictors of apCR. The Rad-score OR of 4.73 represents the odds increase per one standard deviation increase in the Rad-score (SD = 0.55 in the training set). To convert to a per-unit OR: the standardized coefficient βSD = ln(4.73) = 1.554; the per-unit coefficient βunit = βSD/SD = 1.554/0.55 = 2.826; thus the per-unit OR = exp(2.826) = 16.9. This large per-unit OR reflects the narrow standard deviation of the Rad-score distribution and indicates that each unit increase in the Rad-score (spanning approximately 1.8 SD) corresponds to a substantial change in predicted probability. HER2 status and Ki-67 were retained in the model despite not reaching conventional significance (P = 0.101 and P = 0.154, respectively), based on their strong biological rationale and contribution to the clinical model.

### Model diagnostics and risk stratification

**Figure 10:**
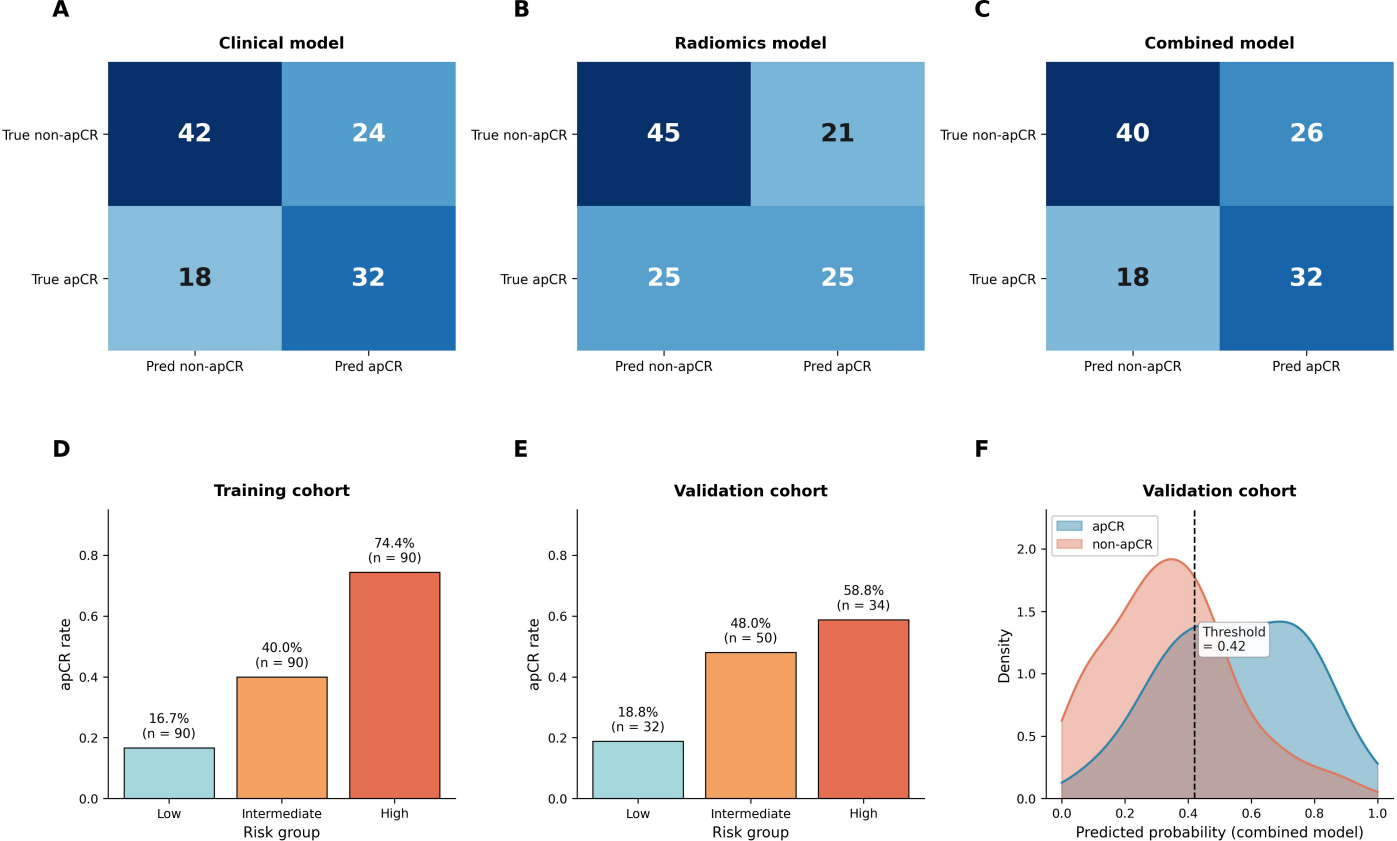
Model diagnostics and risk stratification. **(A)** Validation set confusion matrix for the clinical model at threshold 0.42. **(B)** Radiomics model. **(C)** Combined model. **(D)** Risk stratification in the training cohort by combined model predicted probability tertiles. **(E)** Risk stratification in the validation cohort. **(F)** Predicted probability density distributions by cohort and apCR status; the dashed vertical line indicates the 0.42 classification threshold.

**Figure 10** shows the validation set confusion matrices for all three models (panels A–C) and the risk stratification results (panels D–F). The combined model correctly classified 72 of 116 patients (accuracy 62.1%), with sensitivity of 64.0% and specificity of 60.6%. Of the 58 patients predicted to achieve apCR, 26 (44.8%) were misclassified: they were predicted to be responders but actually had residual axillary disease (1 − PPV = 44.8%). If the combined model were used to guide de-escalation from ALND to SLNB, these 26 patients would have been inappropriately triaged away from complete axillary dissection despite harboring residual disease. This misclassification rate far exceeds the benchmark set by the ACOSOG Z1071 trial, which established that the SLNB false-negative rate (defined as the proportion of patients with residual nodal disease who are incorrectly identified as node-negative by SLNB) should not exceed 10% for acceptable post-NAC axillary staging (8). Although the model’s 1 − PPV and the SLNB false-negative rate are not directly comparable metrics—the former represents the positive misclassification proportion of the prediction model, while the latter represents the diagnostic miss rate of a surgical procedure—both quantify the risk that patients with residual disease are undertreated, and both must be minimized to ensure patient safety. Among the 58 patients predicted as non-apCR, 18 (31.0%) were false negatives who achieved apCR but would have undergone unnecessary ALND under the model.

Risk stratification by training-set-derived tertiles demonstrated a monotonic gradient in observed apCR rates across the three risk groups in both cohorts (**Figures 10D, E**). In the training set, apCR rates were 16.7% (low, n = 90), 40.0% (intermediate, n = 90), and 74.4% (high, n = 90). In the validation set, the corresponding rates were 18.8% (low, n = 32), 48.0% (intermediate, n = 50), and 58.8% (high, n = 34). The attenuation of the high-risk group apCR rate from 74.4% to 58.8% across cohorts reflects the calibration slope shrinkage noted above. The predicted probability density distributions (**Figure 10F**) show substantial overlap between apCR and non-apCR groups, confirming the model cannot reliably dichotomize patients at any single threshold.

### Threshold sensitivity analysis

**Figure 11:**
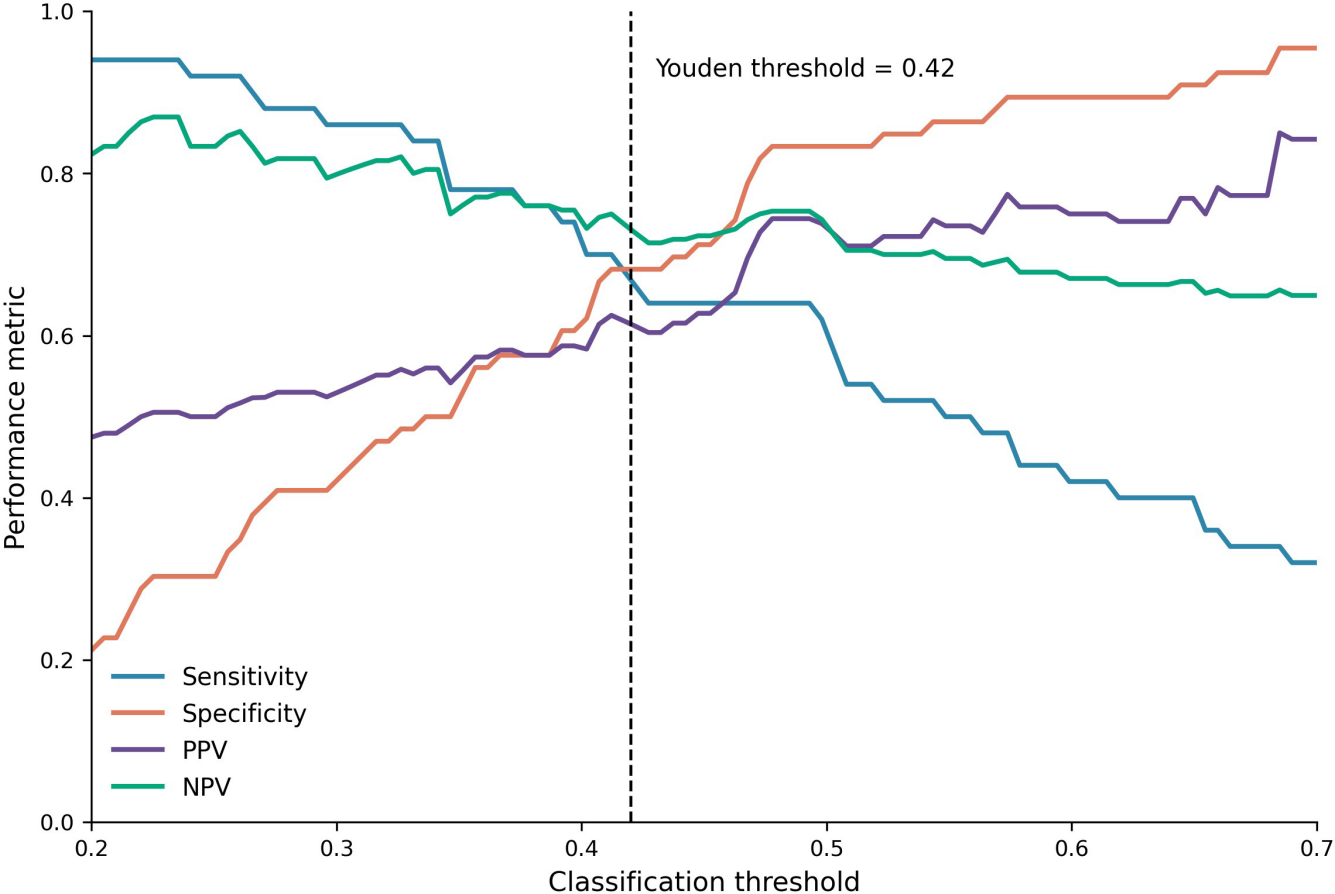
Threshold sensitivity analysis for the combined model in the validation cohort. Sensitivity, specificity, PPV, and NPV are plotted as functions of the classification threshold. The dashed vertical line indicates the Youden-optimized threshold of 0.42.

To assess the robustness of model performance to threshold selection, we evaluated the combined model across classification thresholds ranging from 0.30 to 0.55 in the validation set (**Figure 11**). At the Youden-optimized threshold of 0.42, sensitivity was 0.640 and specificity was 0.606. Lowering the threshold to 0.35 increased sensitivity to 0.76 but reduced specificity to 0.48, while raising the threshold to 0.50 yielded sensitivity of 0.44 and specificity of 0.77. The equal-error-rate threshold (where sensitivity equals specificity) occurred at approximately 0.40, yielding balanced performance of 0.63 for both metrics. No threshold achieved a 1 − PPV below 40%, confirming that the model’s positive predictions are insufficiently reliable for surgical de-escalation at any clinically reasonable operating point.

### Subtype-stratified model performance

To assess the generalizability of the combined model across molecular subtypes, we computed subtype-stratified AUCs (**Table 6**). The combined model performed best in the HER2+/HR+ subgroup (training AUC = 0.758, validation AUC = 0.714) and was least discriminative in the TNBC subgroup (training AUC = 0.694, validation AUC = 0.651). The HER2+/HR− subgroup was too small (n = 6 in validation) for reliable AUC estimation. These results suggest moderate heterogeneity in model performance across subtypes, though all subgroup estimates should be interpreted cautiously given the limited sample sizes. Because several subgroups contained few events, the corresponding 95% confidence intervals were wide (**Table 6**); for example, the validation AUC for the HER2+/HR+ subgroup was 0.714 (95% CI, 0.515–0.882) and that for the TNBC subgroup was 0.651 (95% CI, 0.408–0.857), and the HER2+/HR− subgroup was not estimable in validation. Subtype-specific performance should therefore be regarded as exploratory and hypothesis-generating, and these limitations are reiterated in the Discussion.

**Table 6:** Subtype-stratified AUC of the combined model.

Molecular subtype	n (train/val)	Training AUC (95% CI)	Validation AUC (95% CI)
Luminal A	19/8	0.702 (0.462–0.915)*	0.688 (0.312–0.938)*
Luminal B	114/58	0.731 (0.641–0.812)	0.667 (0.523–0.796)
HER2+/HR+	57/26	0.758 (0.637–0.861)	0.714 (0.515–0.882)
HER2+/HR−	19/6	0.689 (0.448–0.893)*	† (n too small)
TNBC	61/18	0.694 (0.565–0.815)	0.651 (0.408–0.857)*
Overall (combined model)	270/116	0.785 (0.728–0.835)	0.703 (0.610–0.792)

*Small sample size; interpret with caution. †Too few events for reliable AUC estimation. Subtype-specific performance should be regarded as exploratory and hypothesis-generating. Abbreviations as in **Tables 1**, **2**.

## Discussion

**Figure 12:**
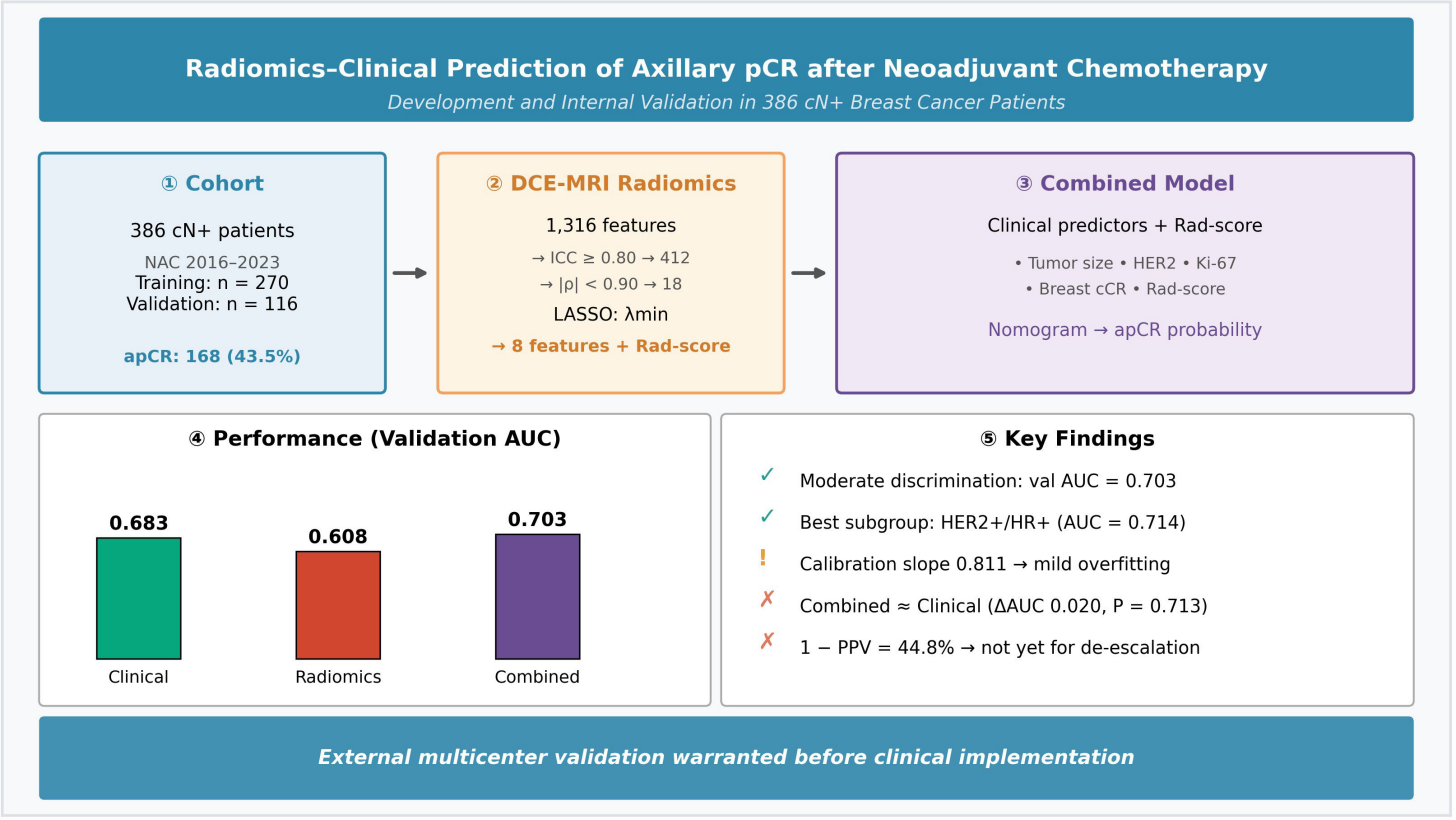
Schematic overview of study design and key results. A total of 386 cN+ breast cancer patients were enrolled (training, n = 270; validation, n = 116). Pre-NAC DCE-MRI radiomic features were extracted and reduced to an 8-feature Rad-score via LASSO. The combined model (clinical predictors + Rad-score) achieved a validation AUC of 0.703, significantly outperforming the radiomics-only model (AUC = 0.608, P = 0.004) but not the clinical-only model (AUC = 0.683, P = 0.713). Calibration slope was 0.811, indicating mild overfitting, and the 1 − PPV of 44.8% precludes clinical use for surgical de-escalation. External multicenter validation is warranted.

In this single-center retrospective study of 386 cN+ breast cancer patients treated with NAC, we developed and internally validated a combined radiomics-clinical prediction model for axillary pathological complete response (**Figure 12**). The combined model achieved a validation AUC of 0.703, calibration slope of 0.811 with intercept of 0.018, and Brier Skill Score of 0.116. While the combined model significantly outperformed the radiomics-only model in the validation cohort (ΔAUC = 0.094, P = 0.004), it did not significantly outperform the clinical-only model (ΔAUC = 0.020, P = 0.713). Quantitatively, the incremental discrimination of the Rad-score over the clinical model was small and its 95% confidence interval crossed zero (ΔAUC = 0.020, 95% CI −0.087 to 0.127, P = 0.713); thus the incremental value of radiomics over established clinical predictors was not statistically confirmed in internal validation, even though the Rad-score carried the largest standardized effect within the combined model (adjusted OR = 4.73 per SD). This finding suggests that, in our cohort, the incremental discriminatory value of the Rad-score over established clinical predictors was modest and not statistically confirmed in the validation set, contrasting with the more promising training-set results. Our overall apCR rate of 43.5% in cN+ patients is consistent with published figures from large institutional series and meta-analyses, which report rates of 35–55% depending on molecular subtype composition and NAC regimens employed (3, 4, 31). The AUC of 0.703 for the combined model falls within the range of 0.65–0.80 reported by comparable radiomics-based studies predicting breast or axillary pCR (20, 32, 33). Liu and colleagues (32) reported a combined radiomics-clinical model achieving an AUC of 0.82 for breast pCR prediction in 326 patients; however, that study focused on breast pCR rather than the more clinically actionable endpoint of axillary pCR. Santucci and colleagues (20) developed an MRI-based radiomics model for axillary response with AUC 0.76 in internal validation, but with a smaller sample and without formal calibration assessment. Our study adds to this literature by providing a comprehensive evaluation framework including calibration slope and intercept, Brier Skill Score, decision curve analysis, threshold sensitivity analysis, and subtype-stratified performance, which are increasingly recognized as essential components of prediction model reporting (25, 26).

A notable finding was the substantial performance degradation of the radiomics-only model from training (AUC = 0.735) to validation (AUC = 0.608), a drop of 0.127 points that was not observed to the same degree for the clinical model (ΔAUC = 0.016). This pattern is characteristic of overfitting in radiomics studies and has been documented across multiple imaging domains (23, 24). The validation calibration slope of 0.811 for the combined model further confirms this overfitting tendency, as a slope below 1.0 indicates systematic overconfidence in extreme predictions. The optimism-corrected training AUCs reported above (0.742 combined, 0.661 radiomics-only, 0.651 clinical-only) reinforce this interpretation, as the radiomics-only model showed the largest optimism (0.074). Several factors may have contributed. First, the LASSO selection at λmin rather than the more conservative λ1se may have retained features with marginal signal; our sensitivity analysis with λ1se (7 features, validation AUC = 0.691) showed only modest performance reduction, suggesting partial feature redundancy. Second, the absence of nested cross-validation means the training AUC of 0.735 for the radiomics model likely overestimates true model performance; a portion of the 0.127-point ΔAUC may reflect optimistic bias in the training estimate rather than genuine external prediction failure. Future studies should employ nested cross-validation or.632+ bootstrap estimation to provide optimism-adjusted training AUC (34). Third, the distributional shift in TNBC prevalence between training (22.6%) and validation (15.5%) cohorts may have altered the feature-outcome relationships, as TNBC tumors are known to exhibit distinct radiomic phenotypes with higher texture heterogeneity (35). To assess whether this imbalance materially affected performance, we repeated the validation analysis after inverse-probability weighting the validation cohort to the training-set molecular-subtype distribution; the weighted validation AUC was 0.699 (versus 0.703 unweighted), indicating that the TNBC distributional shift alone did not fully account for the observed performance attenuation. Finally, we note that the candidate pool reduction from 1,316 to 18 features through ICC filtering and correlation-based pruning was steep (412 features survived ICC ≥ 0.80, and 18 survived the correlation threshold of |ρ| ≥ 0.90). This aggressive pruning, while reducing multicollinearity, may have eliminated features with genuine predictive signal, and the resulting EPV of 6.6 during the LASSO screening phase falls below the recommended minimum of 10 (30). These methodological limitations underscore the importance of external validation.

The nomogram analysis revealed that the Rad-score contributed approximately 61% of the total point allocation (100 of 164 maximum points), with the four clinical variables collectively accounting for only 39%. This imbalance means the nomogram functions essentially as a Rad-score with minor clinical adjustments. If the radiomics signature proves unstable in external datasets—as our own validation data suggest—the entire nomogram becomes unreliable. Furthermore, HER2 status and Ki-67 did not achieve statistical significance in the combined model (P = 0.101 and P = 0.154, respectively) and contributed relatively small point allocations (12 and 10 points, respectively). A simpler model retaining only tumor size, breast cCR, and Rad-score may offer equivalent or better parsimony, and we encourage future investigators to evaluate simplified alternatives. The omission of molecular subtype as an independent variable deserves additional comment. Although univariate analysis demonstrated a significant association between Luminal versus non-Luminal status and apCR (OR = 0.48, P = 0.003), the four-level subtype variable did not achieve independent significance in multivariate analysis, likely due to collinearity with HER2 and Ki-67. Subtype-stratified AUC analysis (**Table 6**) revealed moderate heterogeneity, with validation AUCs ranging from 0.651 (TNBC) to 0.714 (HER2+/HR+), suggesting that subtype-specific models might outperform the pooled approach. Given the established biological importance of molecular subtype in determining chemosensitivity (4, 36), future multicenter studies with larger sample sizes should formally incorporate subtype-stratified analyses and potentially develop subtype-specific models.

From a clinical decision-making perspective, the combined model in its current form cannot be recommended for guiding surgical de-escalation from ALND to SLNB. In the validation set, among 58 patients predicted to achieve apCR, 26 (1 − PPV = 44.8%) actually had residual axillary disease. This misclassification rate substantially exceeds the acceptable threshold for clinical decision-making. The ACOSOG Z1071 trial established that the SLNB false-negative rate should not exceed approximately 10% for safe post-NAC axillary staging (8), and while the model’s 1 − PPV and the SLNB false-negative rate measure different quantities (positive predictive failure of a statistical model versus diagnostic miss rate of a surgical procedure), both ultimately quantify the proportion of patients with residual disease who would be undertreated. Even in the highest tertile, the validation apCR rate was only 58.8%, meaning 41.2% of patients classified as likely responders still harbored residual axillary disease. The threshold sensitivity analysis demonstrated that no classification threshold achieved acceptable positive predictive performance, with 1 − PPV remaining above 37% across the entire threshold range. The decision curve analysis, while showing positive net benefit at threshold probabilities of 20–45%, does not override these safety concerns when the clinical consequence of a false-positive prediction is under-treatment of residual axillary disease. We therefore propose that the model may serve a complementary role in risk communication, patient counseling, or enrichment of clinical trial populations for axillary de-escalation studies, rather than as a standalone decision tool (37, 38).

This study has several important limitations that warrant careful consideration. First, this is a single-center retrospective study, and the internal validation (random split) does not substitute for true external validation across independent institutions with different scanner vendors, acquisition protocols, and patient populations. The absence of ComBat harmonization or phantom-based stability testing means the Rad-score’s transferability to scanners other than the Siemens MAGNETOM Skyra and Prisma platforms used in our study remains entirely unknown (23, 24). Prospective multi-vendor data collection with ComBat feature harmonization and phantom-based stability testing will therefore be a prerequisite for establishing cross-scanner reproducibility before any external application of the Rad-score. Second, the 24.6% exclusion rate raises concerns about selection bias, as exclusion for suboptimal image quality and non-mass-like enhancement may have selected for tumors with more favorable segmentation characteristics, potentially inflating radiomics model performance in ways that would not generalize to unselected clinical populations. Third, the 8-year enrollment period (2016–2023) spans substantial changes in NAC regimens, including the routine adoption of pertuzumab-based dual HER2 blockade and carboplatin-containing regimens for TNBC. Although a formal time-stratified analysis (e.g., 2016–2019 versus 2020–2023) was not performed due to insufficient events per stratum after splitting, this temporal heterogeneity may have contributed to both within-cohort variability and the observed validation performance degradation. We acknowledge this as a limitation and recommend that future studies with larger samples incorporate time-period interaction terms or conduct epoch-stratified sensitivity analyses. Fourth, while we reported ICC > 0.80 for inter-reader feature agreement, we did not perform test-retest stability analysis, which would be necessary to quantify feature robustness to within-patient imaging variability. Fifth, the study was underpowered for meaningful subtype-stratified analyses, particularly for the HER2+/HR− subgroup (n = 25 overall, n = 6 in validation), and the *post hoc* sample size assessment confirmed that the candidate screening phase operated at an EPV of 6.6, below recommended minimums (30). As reported in **Table 6**, the 95% confidence intervals for subgroup AUCs were wide (e.g., validation HER2+/HR+ AUC 0.714 (95% CI 0.515–0.882), TNBC AUC 0.651 (95% CI 0.408–0.857), and the HER2+/HR− subgroup was not estimable in validation), and these subtype-specific findings should therefore be regarded as exploratory and hypothesis-generating.

In summary, this study provides a development and internal validation framework for a combined radiomics-clinical model predicting axillary pCR after NAC in cN+ breast cancer patients. The transparent reporting of both positive and negative results—notably, the finding that the combined model did not significantly outperform clinical predictors alone in the validation set—reflects our commitment to rigorous prediction model research and avoidance of overoptimistic claims that have been a pervasive concern in the radiomics literature (39, 40). Several methodological improvements should be incorporated in future work: nested cross-validation or.632+ bootstrap for bias-corrected training performance estimates; ComBat harmonization or feature stability testing for multi-scanner applicability; formal time-period stratification to address treatment era effects; prospective data collection with *a priori* sample size estimation based on Riley and colleagues (30); and multi-center external validation following TRIPOD Type 3 guidelines (22). Integration of additional data modalities, such as ultrasound-based axillary features, circulating tumor DNA, or tumor-infiltrating lymphocyte scores, may also improve model discrimination and clinical relevance (41, 42). The model should be regarded as a research tool requiring external multicenter validation before any clinical implementation.

Beyond imaging modalities, complementary genomic biomarkers identified through pan-cancer analyses also hold promise. For example, PPME1 and BEND3 have been associated with immune regulation and prognosis in breast cancer. Similarly, CLIC6 and CORO1A have shown correlations with tumor immune infiltration and patient outcomes (43–46). Although these genomic markers differ methodologically from our radiomics-based approach, they underscore a broader scientific consensus: robust, clinically actionable biomarkers require rigorous validation across independent cohorts. Future research may benefit from integrating multi-omics data—including radiomic features, genomic signatures, and clinical variables—to improve predictive performance and generate deeper biological insight.

## Conclusions

A combined radiomics-clinical model incorporating tumor size, HER2 status, Ki-67, breast clinical complete response, and an MRI-derived Rad-score demonstrated moderate discrimination (validation AUC = 0.703) for predicting axillary pathological complete response in cN+ breast cancer patients after neoadjuvant chemotherapy. The Rad-score was the strongest independent predictor (adjusted OR = 4.73 per 1 SD, corresponding to a per-unit OR of 16.9, P < 0.001). However, the combined model did not significantly outperform the clinical-only model in the validation cohort (P = 0.713), and calibration metrics indicate moderate overfitting (validation slope = 0.811, intercept = 0.018). The model in its current form cannot support clinical decision-making for surgical de-escalation, and external multicenter validation with prospective design is warranted. This work should be considered a model development study rather than a validation study.

## Data Availability

The raw data supporting the conclusions of this article will be made available by the authors, without undue reservation.

## References

[1] Cortazar P Zhang L Untch M Mehta K Costantino JP Wolmark N . Pathological complete response and long-term clinical benefit in breast cancer: the CTNeoBC pooled analysis. Lancet (Lond Engl). (2014) 384:164–72. doi: 10.1016/S0140-6736(13)62422-8 24529560

[2] Spring LM Fell G Arfe A Sharma C Greenup R Reynolds KL . Pathologic complete response after neoadjuvant chemotherapy and impact on breast cancer recurrence and survival: a comprehensive meta-analysis. Clin Cancer Res: Off J Am Assoc For Cancer Res. (2020) 26:2838–48. doi: 10.1158/1078-0432.CCR-19-3492 32046998 PMC7299787

[3] Boughey JC McCall LM Ballman KV Mittendorf EA Ahrendt GM Wilke LG . Tumor biology correlates with rates of breast-conserving surgery and pathologic complete response after neoadjuvant chemotherapy for breast cancer: findings from the ACOSOG Z1071 (Alliance) prospective multicenter clinical trial. Ann Surg. (2014) 260:608–16. doi: 10.1097/SLA.0000000000000924 25203877 PMC4159769

[4] von Minckwitz G Untch M Blohmer J-U Costa SD Eidtmann H Fasching PA . Definition and impact of pathologic complete response on prognosis after neoadjuvant chemotherapy in various intrinsic breast cancer subtypes. J Clin Oncol Off J Am Soc Clin Oncol. (2012) 30:1796–804. doi: 10.1200/JCO.2011.38.8595 22508812

[5] DiSipio T Rye S Newman B Hayes S . Incidence of unilateral arm lymphoedema after breast cancer: a systematic review and meta-analysis. Lancet Oncol. (2013) 14:500–15. doi: 10.1016/S1470-2045(13)70076-7 23540561

[6] Lucci A McCall LM Beitsch PD Whitworth PW Reintgen DS Blumencranz PW . Surgical complications associated with sentinel lymph node dissection (SLND) plus axillary lymph node dissection compared with SLND alone in the American College of Surgeons Oncology Group Trial Z0011. J Clin Oncol Off J Am Soc Clin Oncol. (2007) 25:3657–63. doi: 10.1200/jco.2006.07.4062 17485711

[7] Classe J-M Bordes V Campion L Mignotte H Dravet F Leveque J . Sentinel lymph node biopsy after neoadjuvant chemotherapy for advanced breast cancer: results of Ganglion Sentinelle et Chimiotherapie Neoadjuvante, a French prospective multicentric study. J Clin Oncol Off J Am Soc Clin Oncol. (2008) 27:726–32. doi: 10.1200/JCO.2008.18.3228 19114697

[8] Boughey JC Suman VJ Mittendorf EA Ahrendt GM Wilke LG Taback B . Sentinel lymph node surgery after neoadjuvant chemotherapy in patients with node-positive breast cancer: the ACOSOG Z1071 (Alliance) clinical trial. JAMA. (2013) 310:1455–61. doi: 10.1001/jama.2013.278932 24101169 PMC4075763

[9] Kuehn T Bauerfeind I Fehm T Fleige B Hausschild M Helms G . Sentinel-lymph-node biopsy in patients with breast cancer before and after neoadjuvant chemotherapy (SENTINA): a prospective, multicentre cohort study. Lancet Oncol. (2013) 14:609–18. doi: 10.1016/S1470-2045(13)70166-9 23683750

[10] Boileau J-F Poirier B Basik M Holloway CMB Gaboury L Sideris L . Sentinel node biopsy after neoadjuvant chemotherapy in biopsy-proven node-positive breast cancer: the SN FNAC study. J Clin Oncol Off J Am Soc Clin Oncol. (2014) 33:258–64. doi: 10.1200/JCO.2014.55.7827 25452445

[11] Mamounas EP Anderson SJ Dignam JJ Bear HD Julian TB Geyer CE . Predictors of locoregional recurrence after neoadjuvant chemotherapy: results from combined analysis of National Surgical Adjuvant Breast and Bowel Project B-18 and B-27. J Clin Oncol Off J Am Soc Clin Oncol. (2012) 30:3960–6. doi: 10.1200/JCO.2011.40.8369 23032615 PMC3488269

[12] Hwang HW Jung H Hyeon J Park YH Ahn JS Im Y-H . A nomogram to predict pathologic complete response (pCR) and the value of tumor-infiltrating lymphocytes (TILs) for prediction of response to neoadjuvant chemotherapy (NAC) in breast cancer patients. Breast Cancer Res Treat. (2018) 173:255–66. doi: 10.1007/s10549-018-4981-x 30324273

[13] Kim S-Y Cho N Choi Y Lee SH Ha SM Kim ES . Factors affecting pathologic complete response following neoadjuvant chemotherapy in breast cancer: development and validation of a predictive nomogram. Radiology. (2021) 299:290–300. doi: 10.1148/radiol.2021203871 33754824

[14] Tadros AB Yang WT Krishnamurthy S Rauch GM Smith BD Valero V . Identification of patients with documented pathologic complete response in the breast after neoadjuvant chemotherapy for omission of axillary surgery. JAMA Surg. (2017) 152:665–70. doi: 10.1001/jamasurg.2017.0562 28423171 PMC5547923

[15] Dialani V Chadashvili T Slanetz PJ . Role of imaging in neoadjuvant therapy for breast cancer. Ann Surg Oncol. (2015) 22:1416–24. doi: 10.1245/s10434-015-4403-9 25727555

[16] Rauch GM Adrada BE Kuerer HM van la Parra RFD Leung JWT Yang WT . Multimodality imaging for evaluating response to neoadjuvant chemotherapy in breast cancer. AJR Am J Roentgenol. (2016) 208:290–9. doi: 10.2214/AJR.16.17223 27809573

[17] Lambin P Leijenaar RTH Deist TM Peerlings J de Jong EEC van Timmeren J . Radiomics: the bridge between medical imaging and personalized medicine. Nat Rev Clin Oncol. (2017) 14:749–62. doi: 10.1038/nrclinonc.2017.141 28975929

[18] Gillies RJ Kinahan PE Hricak H . Radiomics: images are more than pictures, they are data. Radiology. (2015) 278:563–77. doi: 10.1148/radiol.2015151169 26579733 PMC4734157

[19] Fan M Li H Wang S Zheng B Zhang J Li L . Radiomic analysis reveals DCE-MRI features for prediction of molecular subtypes of breast cancer. PloS One. (2017) 12:e0171683. doi: 10.1371/journal.pone.0171683 28166261 PMC5293281

[20] Santucci D Faiella E Cordelli E Sicilia R de Felice C Zobel BB . 3T MRI-radiomic approach to predict for lymph node status in breast cancer patients. Cancers. (2021) 13:2228. doi: 10.3390/cancers13092228 34066451 PMC8124168

[21] Braman NM Etesami M Prasanna P Dubchuk C Gilmore H Tiwari P . Intratumoral and peritumoral radiomics for the pretreatment prediction of pathological complete response to neoadjuvant chemotherapy based on breast DCE-MRI. Breast Cancer Res: BCR. (2017) 19:57. doi: 10.1186/s13058-017-0846-1 28521821 PMC5437672

[22] Collins GS Reitsma JB Altman DG Moons KGM . Transparent reporting of a multivariable prediction model for individual prognosis or diagnosis (TRIPOD): the TRIPOD statement. BMC Med. (2015) 13:1. doi: 10.1186/s12916-014-0241-z 25563062 PMC4284921

[23] Zwanenburg A Vallières M Abdalah MA Aerts HJWL Andrearczyk V Apte A . The Image Biomarker Standardization Initiative: standardized quantitative radiomics for high-throughput image-based phenotyping. Radiology. (2020) 295:328–38. doi: 10.1148/radiol.2020191145 32154773 PMC7193906

[24] Traverso A Wee L Dekker A Gillies R . Repeatability and reproducibility of radiomic features: a systematic review. Int J Radiat Oncol Biol Phys. (2018) 102:1143–58. doi: 10.1016/j.ijrobp.2018.05.053 30170872 PMC6690209

[25] Steyerberg EW Vickers AJ Cook NR Gerds T Gonen M Obuchowski N . Assessing the performance of prediction models: a framework for traditional and novel measures. Epidemiol (Cambridge Mass). (2010) 21:128–38. doi: 10.1097/EDE.0b013e3181c30fb2 20010215 PMC3575184

[26] Van Calster B McLernon DJ van Smeden M Wynants L Steyerberg EW . Calibration: the Achilles heel of predictive analytics. BMC Med. (2019) 17:230. doi: 10.1186/s12916-019-1466-7 31842878 PMC6912996

[27] van Griethuysen JJM Fedorov A Parmar C Hosny A Aucoin N Narayan V . Computational radiomics system to decode the radiographic phenotype. Cancer Res. (2017) 77:e104–7. doi: 10.1158/0008-5472.CAN-17-0339 29092951 PMC5672828

[28] DeLong ER DeLong DM Clarke-Pearson DL . Comparing the areas under two or more correlated receiver operating characteristic curves: a nonparametric approach. Biometrics. (1988) 44:837–48. doi: 10.2307/2531595 3203132

[29] Vickers AJ Elkin EB . Decision curve analysis: a novel method for evaluating prediction models. Med Decis Mak: Int J Soc For Med Decis Mak. (2006) 26:565–74. doi: 10.1177/0272989x06295361 17099194 PMC2577036

[30] Riley RD Ensor J Snell KIE Harrell FE Martin GP Reitsma JB . Calculating the sample size required for developing a clinical prediction model. BMJ (Clin Res ed). (2020) 368:m441. doi: 10.1136/bmj.m441 32188600

[31] Mougalian SS Soulos PR Killelea BK Lannin DR Abu-Khalaf MM DiGiovanna MP . Use of neoadjuvant chemotherapy for patients with stage I to III breast cancer in the United States. Cancer. (2015) 121:2544–52. doi: 10.1002/cncr.29348 25902916

[32] Liu Z Li Z Qu J Zhang R Zhou X Li L . Radiomics of multiparametric MRI for pretreatment prediction of pathologic complete response to neoadjuvant chemotherapy in breast cancer: a multicenter study. Clin Cancer Res: Off J Am Assoc For Cancer Res. (2019) 25:3538–47. doi: 10.1158/1078-0432.CCR-18-3190 30842125

[33] Bitencourt AGV Gibbs P Rossi Saccarelli C Daimiel I Lo Gullo R Fox MJ . MRI-based machine learning radiomics can predict HER2 expression level and pathologic response after neoadjuvant therapy in HER2 overexpressing breast cancer. EBioMedicine. (2020) 61:103042. doi: 10.1016/j.ebiom.2020.103042 33039708 PMC7648120

[34] Moons KGM Wolff RF Riley RD Whiting PF Westwood M Collins GS . PROBAST: a tool to assess risk of bias and applicability of prediction model studies: explanation and elaboration. Ann Internal Med. (2019) 170:W1–W33. doi: 10.7326/M18-1377 30596876

[35] Li H Zhu Y Burnside ES Huang E Drukker K Hoadley KA . Quantitative MRI radiomics in the prediction of molecular classifications of breast cancer subtypes in the TCGA/TCIA data set. NPJ Breast Cancer. (2016) 2:16012. doi: 10.1038/npjbcancer.2016.12 27853751 PMC5108580

[36] Prat A Fan C Fernández A Hoadley KA Martinello R Vidal M . Response and survival of breast cancer intrinsic subtypes following multi-agent neoadjuvant chemotherapy. BMC Med. (2015) 13:303. doi: 10.1186/s12916-015-0540-z 26684470 PMC4683815

[37] Tadros AB Wen HY Morrow M . Breast cancers of special histologic subtypes are biologically diverse. Ann Surg Oncol. (2018) 25:3158–64. doi: 10.1245/s10434-018-6687-z 30094484 PMC6128764

[38] Giuliano AE Ballman KV McCall L Beitsch PD Brennan MB Kelemen PR . Effect of axillary dissection vs no axillary dissection on 10-year overall survival among women with invasive breast cancer and sentinel node metastasis: the ACOSOG Z0011 (Alliance) randomized clinical trial. JAMA. (2017) 318:918–26. doi: 10.1001/jama.2017.11470 28898379 PMC5672806

[39] Park JE Kim D Kim HS Park SY Kim JY Cho SJ . Quality of science and reporting of radiomics in oncologic studies: room for improvement according to radiomics quality score and TRIPOD statement. Eur Radio. (2019) 30:523–36. doi: 10.1007/s00330-019-06360-z 31350588

[40] Chalkidou A O'Doherty MJ Marsden PK . False discovery rates in PET and CT studies with texture features: a systematic review. PloS One. (2015) 10:e0124165. doi: 10.1371/journal.pone.0124165 25938522 PMC4418696

[41] Garcia-Murillas I Schiavon G Weigelt B Ng C Hrebien S Cutts RJ . Mutation tracking in circulating tumor DNA predicts relapse in early breast cancer. Sci Transl Med. (2015) 7:302ra133. doi: 10.1126/scitranslmed.aab0021 26311728

[42] Denkert C von Minckwitz G Darb-Esfahani S Lederer B Heppner BI Weber KE . Tumour-infiltrating lymphocytes and prognosis in different subtypes of breast cancer: a pooled analysis of 3771 patients treated with neoadjuvant therapy. Lancet Oncol. (2017) 19:40–50. doi: 10.1016/S1470-2045(17)30904-X 29233559

[43] Wang Y Zhang Y Li Y Ma H Zhao J Ismtula D . The value of protein phosphatase methylesterase 1 in diagnosis, prognosis and immunoregulation: from pan-cancer analysis to breast cancer verification. Front Immunol. (2026) 17:1770711. doi: 10.3389/fimmu.2026.1770711 41884849 PMC13008989

[44] Gou Y Li Y Wang Y Ma H Li H Geni R . Integrated pan-cancer profiling and breast cancer validation identify BEND3 as a potential prognostic and immune biomarker. Breast Cancer (Dove Med Press). (2025) 17:1439–61. doi: 10.2147/BCTT.S553681 41497890 PMC12766032

[45] Wang J Wang Y Ma H Li Y Hou J Li J . CLIC6's role in cancer: from broad analysis to breast cancer validation. Front Oncol. (2025) 15:1667589. doi: 10.3389/fonc.2025.1667589 41142627 PMC12545143

[46] Elihamu D Li Y Wang Y Cui H Xing Y Peng H . CORO1A: a pan-cancer prognosis, diagnostic and immune biomarker based on breast cancer validation. Front Oncol. (2025) 15:1670526. doi: 10.3389/fonc.2025.1670526 41122645 PMC12535887

