# Supplementary material for: Development and internal validation of a radiomics-clinical combined model for predicting axillary pathological complete response in clinically node-positive breast cancer patients after neoadjuvant chemotherapy
Source: Front Oncol. 2026 Jul 10;16:1876446. doi: 10.3389/fonc.2026.1876446 (PMC13395862; doi:10.3389/fonc.2026.1876446)
Supplement: Supplementary file 1 [file Table1.docx]

**Supplementary Tables**

**Supplementary Table S1. Rad-score formula: selected radiomic features and their LASSO coefficients.**

| **Rank** | **Feature** | **LASSO coefficient (unstandardized)** | **Feature class** |
| --- | --- | --- | --- |
| 1 | Wavelet-LHL_GLRLM_RunEntropy | −0.337 | Texture (GLRLM) |
| 2 | Shape_Sphericity | +0.270 | Shape |
| 3 | Wavelet-HHL_GLSZM_ZonePercentage | −0.215 | Texture (GLSZM) |
| 4 | LoG-3mm_Firstorder_Skewness | +0.184 | First-order |
| 5 | Wavelet-HLL_GLDM_DependenceEntropy | −0.158 | Texture (GLDM) |
| 6 | Original_NGTDM_Contrast | +0.142 | Texture (NGTDM) |
| 7 | Wavelet-LLH_GLCM_Correlation | −0.098 | Texture (GLCM) |
| 8 | Wavelet-HLH_GLCM_Imc1 | −0.017 | Texture (GLCM) |
|  | Intercept | −0.186 |  |

*Rad-score = Σ (coefficient × feature value) + intercept. Features are reported in unstandardized scale. Abbreviations: GLCM, gray-level co-occurrence matrix; GLRLM, gray-level run-length matrix; GLSZM, gray-level size zone matrix; GLDM, gray-level dependence matrix; NGTDM, neighboring gray-tone difference matrix; LoG, Laplacian of Gaussian.*

**Supplementary Table S2. TRIPOD checklist for prediction model development and internal validation (Type 2).**

| **Item** | **Checklist item** | **Reported** |
| --- | --- | --- |
| Title and abstract |  |  |
| 1 | Identify study as developing/validating a prediction model | Title, Abstract |
| 2 | Provide summary of objectives, study design, methods, results, conclusions | Abstract |
| Introduction |  |  |
| 3a | Explain medical context and rationale | Introduction |
| 3b | Specify objectives (development, validation, comparison) | Introduction |
| Methods |  |  |
| 4a | Study design or data source | Methods: Study Design |
| 4b | Key study dates | Methods: Study Design |
| 5a | Study setting | Methods: Study Design |
| 5b | Eligibility criteria for participants | Methods: Study Design |
| 5c | Treatments received | Methods: Clinical Data |
| 6a | Outcome definition | Methods: Endpoint |
| 6b | Actions to blind outcome assessment | Methods: Radiomics Pipeline |
| 7a | Candidate predictors, definitions, measurement | Methods: Clinical Data, Radiomics |
| 7b | Actions to blind predictor assessment | Methods: Radiomics Pipeline |
| 8 | Sample size | Methods: Sample Size Considerations |
| 9 | Missing data handling | Methods: Clinical Data |
| 10a | Methods for predictor handling | Methods: Clinical Predictor Selection |
| 10b | Model-building procedures | Methods: Feature Selection, Model Construction |
| 10d | Specify model performance measures | Methods: Model Construction |
| 10e | Describe model update/comparison | Methods: Model Construction |
| 11 | Risk groups | Methods: Model Construction |
| Results |  |  |
| 13a | Participant flow (exclusions) | Results: Figure 1, Baseline |
| 13b | Participant characteristics | Table 1 |
| 14a | Number of events and predictors | Methods: Sample Size |
| 14b | Unadjusted associations | Table 2 |
| 15a | Final model predictors | Tables 3, S1 |
| 15b | Present model for individualized prediction | Figure 8 (Nomogram) |
| 16 | Model performance | Table 4, Figures 5–7 |
| 17 | Model comparison | Table 5 |
| Discussion |  |  |
| 18 | Limitations (non-representativeness, missing data, small sample) | Discussion |
| 19a | Interpretation considering study objectives | Discussion |
| 19b | Overall interpretation | Discussion, Conclusion |
| 20 | Potential clinical use and implications for research | Discussion, Conclusion |
| Other |  |  |
| 21 | Supplementary resources | Supplementary Tables S1–S4 |
| 22 | Study funding and role of funders | Declarations |

*TRIPOD Type 2: development and internal validation using resampling or split-sample methods. Abbreviations: TRIPOD, Transparent Reporting of a multivariable prediction model for Individual Prognosis Or Diagnosis.*

**Supplementary Table S3. Radiomics Quality Score (RQS) self-assessment.**

| **Item** | **Criterion** | **Max score** | **Score** | **Evidence** |
| --- | --- | --- | --- | --- |
| 1 | Image protocol quality: well-documented acquisition | +1 | +1 | Methods: MRI Acquisition |
| 2 | Multiple segmentations to test robustness | +1 | +1 | ICC assessment between 2 readers |
| 3 | Phantom study on all scanners | +1 | 0 | Not performed (limitation) |
| 4 | Imaging at multiple time points | +1 | 0 | Single pre-NAC time point only |
| 5 | Feature reduction / multiple testing correction | +3 | +3 | ICC + correlation filter + LASSO |
| 6 | Multivariable analysis with non-radiomic features | +1 | +1 | Combined model constructed |
| 7 | Detect and discuss biological correlates | +1 | 0 | Not addressed in depth |
| 8 | Cut-off analyses | +1 | +1 | Threshold sensitivity analysis |
| 9 | Discrimination statistics (e.g., AUC) | +2 | +2 | AUC with bootstrap 95% CI |
| 10 | Calibration statistics | +2 | +2 | Slope, intercept, H-L test, Brier |
| 11 | Prospective study registered | +7 | 0 | Retrospective design |
| 12 | Validation (internal resampling/split) | +2 | +2 | Random 70:30 split validation |
| 13 | Validation on distinct dataset | +3 | 0 | No external validation |
| 14 | Comparison with gold standard | +2 | +2 | Compared with ypN0 pathology |
| 15 | Potential clinical utility | +2 | +2 | Decision curve analysis |
| 16 | Cost-effectiveness analysis | +1 | 0 | Not performed |
| 17 | Open science / data available | +4 | +1 | Code available upon request |
| Total |  | 36 | 17 (47%) |  |

*Total RQS score: 17/36 (47%). Major deficiencies: lack of phantom testing, single time-point imaging, retrospective design, absence of external validation, and no formal cost-effectiveness analysis. These limitations are acknowledged in the Discussion and should be addressed in future prospective multicenter studies.*

**Supplementary Table S4. Sensitivity analysis: performance of the λ1se model (7 features) compared with the primary λmin model (8 features).**

| **Metric** | **λmin model (primary)** | **λ1se model (sensitivity)** | **Δ (λ1se − λmin)** |
| --- | --- | --- | --- |
| Features retained | 8 | 7 | −1 |
| Training AUC (95% CI) | 0.785 (0.728–0.835) | 0.772 (0.714–0.826) | −0.013 |
| Validation AUC (95% CI) | 0.703 (0.610–0.792) | 0.691 (0.596–0.782) | −0.012 |
| Training Brier score | 0.186 | 0.191 | +0.005 |
| Validation Brier score | 0.217 | 0.221 | +0.004 |
| Validation calibration slope | 0.811 | 0.834 | +0.023 |
| Validation calibration intercept | +0.018 | +0.026 | +0.008 |
| Validation Brier Skill Score | 0.116 | 0.100 | −0.016 |

*The λ1se model (one standard error rule) retained 7 features versus 8 in the primary λmin model. Dropping the lowest-weight feature (Wavelet-HLH_GLCM_Imc1; coefficient −0.017) produced only marginal decrements in AUC, supporting the notion that the additional feature contributed little discriminatory information. Abbreviations as in Tables 1 and 4.*

**Supplementary Table S5. DCE-MRI acquisition and reconstruction parameters.**

| **Parameter** | **Value** |
| --- | --- |
| Scanner | 3.0 T Siemens MAGNETOM Skyra / Prisma |
| Coil | Dedicated bilateral breast phased-array coil |
| Sequence | Transverse fat-suppressed 3D T1-weighted gradient-echo (VIBE) |
| Fat suppression | Frequency-selective fat saturation |
| Repetition time (TR) | 4.5–5.2 ms |
| Echo time (TE) | 1.7–2.0 ms |
| Flip angle | 10° |
| Field of view | 320–340 mm |
| Matrix | 384 × 384 |
| Slice thickness | 1.0–1.5 mm (isotropic acquisition) |
| Receiver bandwidth | 380–440 Hz/pixel |
| Parallel imaging | GRAPPA, acceleration factor 2 |
| Contrast agent | Gadoteric acid (Dotarem) |
| Contrast dose | 0.1 mmol/kg body weight |
| Injection rate | 2.0 mL/s (power injector) |
| Saline flush | 20 mL at 2.0 mL/s |
| Dynamic phases | 6 (1 pre-contrast + 5 post-contrast) |
| Temporal resolution | ~60 s per phase (~6 min total) |
| Reconstruction | Vendor-standard online GRAPPA; no additional smoothing/post-filter |
| Resampling (radiomics) | Isotropic 1 × 1 × 1 mm, B-spline interpolation |
| Intensity discretization | Fixed bin width 25 a.u. |

**Supplementary Table S6. Bootstrap stability of the eight LASSO-selected features (1,000 resamples).**

| **Rank** | **Feature** | **LASSO coef.** | **Selection frequency (%)** |
| --- | --- | --- | --- |
| 1 | Wavelet-LHL_GLRLM_RunEntropy | −0.337 | 96 |
| 2 | Shape_Sphericity | +0.270 | 94 |
| 3 | Wavelet-HHL_GLSZM_ZonePercentage | −0.215 | 89 |
| 4 | LoG-3mm_Firstorder_Skewness | +0.184 | 85 |
| 5 | Wavelet-HLL_GLDM_DependenceEntropy | −0.158 | 82 |
| 6 | Original_NGTDM_Contrast | +0.142 | 79 |
| 7 | Wavelet-LLH_GLCM_Correlation | −0.098 | 74 |
| 8 | Wavelet-HLH_GLCM_Imc1 | −0.017 | 71 |

*Each feature was selected in >70% of 1,000 bootstrap resamples of the training set, supporting reproducibility.*

**Supplementary Table S7. Extended combined model including molecular subtype (four-level).**

| **Predictor** | **Adjusted OR (95% CI)** | **P value** |
| --- | --- | --- |
| Tumor size (per mm) | 0.98 (0.97–1.00) | 0.10 |
| HER2-positive | 1.55 (0.82–2.93) | 0.18 |
| Ki-67 (per %) | 1.01 (1.00–1.03) | 0.21 |
| Breast clinical CR | 2.21 (1.21–4.04) | 0.010 |
| Rad-score (per 1 SD) | 4.55 (2.70–7.67) | <0.001 |
| Molecular subtype (4-level) | — | 0.18 (LR test) |

*Adding molecular subtype did not reach significance (likelihood-ratio P = 0.18) and did not improve validation discrimination (AUC 0.703 → 0.706, DeLong P = 0.79); variance-inflation factors for HER2 and Ki-67 exceeded 2.5, indicating collinearity. Subtype was therefore not retained as an independent predictor.*
